# Tensin 1 (*TNS1*) is a modifier gene for low body mass index (BMI) in homozygous [*F508del*]CFTR patients

**DOI:** 10.14814/phy2.14886

**Published:** 2021-06-04

**Authors:** Nathan I. Walton, Xijun Zhang, Anthony R. Soltis, Joshua Starr, Clifton L. Dalgard, Matthew D. Wilkerson, Douglas Conrad, Harvey B. Pollard

**Affiliations:** ^1^ The Collaborative Health Initiative Research Program Uniformed Services University of the Health Sciences Bethesda MD USA; ^2^ Consortium for Health and Military Performance Uniformed Services University of the Health Sciences Bethesda MD USA; ^3^ Henry M. Jackson Foundation for the Advancement of Military Medicine Bethesda MD USA; ^4^ The American Genome Center Uniformed Services University of the Health Sciences Bethesda MD USA; ^5^ Department of Anatomy, Physiology, and Genetics Uniformed Services University of the Health Sciences Bethesda MD USA; ^6^ Department of Medicine University of California San Diego CA USA

**Keywords:** BMI, cystic fibrosis, TNS1, whole genome sequencing

## Abstract

Cystic fibrosis (CF) is a life‐limiting autosomal recessive genetic disease caused by variants in the CFTR gene, most commonly by the *[F508del]* variant. Although CF is a classical Mendelian disease, genetic variants in several modifier genes have been associated with variation of the clinical phenotype for pulmonary and gastrointestinal function and urogenital development. We hypothesized that whole genome sequencing of a well‐phenotyped CF populations might identify novel variants in known, or hitherto unknown, modifier genes. Whole genome sequencing was performed on the Illumina HiSeq X platform for 98 clinically diagnosed cystic fibrosis patient samples from the Adult CF Clinic at the University of California San Diego (UCSD). We compared protein‐coding, non‐silent variants genome wide between CFTR [*F508del*] homozygotes vs CFTR compound heterozygotes. Based on a single variant score test, we found 3 SNPs in common variants (MAF >5%) that occurred at significantly different rates between homozygous [*F508del*]CFTR and compound heterozygous [*F508del*]CFTR patients. The 3 SNPs were all located in one gene on chromosome 2: Tensin 1 (TNS1: rs3796028; rs2571445: and rs918949). We observed significantly lower BMIs in homozygous [*F508del*]CFTR patients who were also homozygous for Tensin 1 rs918949 (T/T) (*p* = 0.023) or rs2571445 (G/G) (*p* = 0.02) variants. The Tensin 1 gene is thus a potential modifier gene for low BMI in CF patients homozygous for the *[F508del]CFTR* variant.

## Graphical abstract

We hypothesized that whole genome sequencing of a well‐phenotyped CF populations might identify novel variants in known, or hitherto unknown, modifier genes. We compared protein‐coding, non‐silent variants genomewide between CFTR [F508del] homozygotes vs CFTR compound heterozygotes. We found that the Tensin 1 gene is a potential modifier gene for low BMI in CF patients homozygous for the [F508del]CFTR variant.

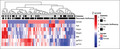

## 1 INTRODUCTION

Cystic fibrosis (CF) is an autosomal recessive genetic disease caused by variants in the cystic fibrosis transmembrane conductance regulator (CFTR) gene (Kerem et al., 1989). CFTR is a membrane bound cAMP‐regulated chloride/bicarbonate channel that is expressed in epithelial cells, and is essential for appropriate mucus function in the sino‐pulmonary and gastro‐intestinal tracts (Bertrand & Frizzel, 2003; Pilewski & Frizzel, 1999). The most prevalent CFTR variant is the deletion of the phenylalanine amino acid at position 508 ([*F508del*]), which is found in ~66% of the CF population (Kerem et al., 1989; Thibodeau et al., 2010). Other disease‐causing CFTR variants create heterozygous genotypes with *[F508del]*, and CF patients with two non‐[*F508del*] variants are also found less frequently (Kerem et al., 1990). The CF patient genotype has been correlated with severity of disease. For example, homozygous [F508del]CFTR patients generally have a more pronounced clinical challenges compared to compound heterozygous [F508del]CFTR and non‐[F508del]CFTR patients (Cystic Fibrosis Genotype‐Phenotype Consortium, 1993; Johansen et al., 1991; McKone et al., 2003, 2006). Compound [*F508del*]CFTR heterozygotes have one F508 deletion as well as another CFTR variant, such as G551D, W1282X, and others (Castellani et al., 2008). Additionally, CF severity, regardless of genotype, can also be impacted by modifier genes and environmental factors. For example, at least 56 non‐CFTR modifier genes have been described which impact selectively and differentially with disease severity, principally in lung, but also in other affected organs (Cutting, 2010; D'Antonio et al., 2019; Knowles & Drumm, 2012; Pollard & Pollard, 2018; Strug et al., 2016). Variants in modifier genes only have functional consequences when the target gene itself has a dysfunctional variant. Furthermore, while variation in disease severity is affected by environmental factors (e.g. airway microbiome, air quality, and socioeconomic driven parameters) (Campbell et al., 1992; Elkins et al., 2006; Ramsay et al., 2016), the majority of phenotype variation is caused by cumulative effects of these modifier genes (Cutting, 2015; Mekus et al., 2000; Vanscoy et al., 2007). It has therefore been suggested that targeting these modifier genes could further benefit the CF patient quality of life (Cutting, 2010; Taylor et al., 2011).

The earliest recognized clinical phenotypes for individuals with CF have included malnutrition and failure to thrive (Kraemer et al., 1978; Pencharz & Durie, 2000; Sinaasappel et al., 2002). This problem results from pancreatic insufficiency due to pancreatic duct obstruction, and current treatment for pancreatic‐insufficient CF patients still includes oral pancreatic enzyme replacement therapy (PERT) (De Lisle & Borowitz, 2013). Although lung health and nutritional status are positively correlated (Stephenson et al., 2013), there is also a fraction of CF patients who do not suffer from malnutrition. In some instances, they have BMIs that indicate an overweight or approaching overweight status, regardless of CFTR mutation class, or pancreatic status (Dray et al., 2005; Kastner‐Cole et al., 2005). These outliers are not fully explained by residual function CFTR variations, suggesting there are unknown BMI modifier genes that play a significant role in CF nutritional status (Bradley et al., 2012).

Potential modifier genes have previously been found by CF genotype‐phenotype association studies (Boelle et al., 2019; Corvol et al., 2015; Drumm et al., 2005; Garred et al., 1999; Weiler & Drumm, 2013; Wright et al., 2011). Many of these studies focus on variations within the homozygous [*F508del*]CFTR genotype, but others include alternative CFTR genotypes as part of their cohorts. Since there is a correlation between CF severity and whether the patient is homozygous [*F508del*]CFTR or compound heterozygous [*F508del*]CFTR, we hypothesized that an association study between these two group could identify variants that may explain distinct pathogenesis based on CFTR group status.

To test this hypothesis, we performed whole genome sequencing (WGS) on a single population of adult CF patients to identify common variants (MAF >5%) that were associated with either homozygous or compound heterozygous *[F508del]*CFTR patients. We found such a non‐CFTR gene, and asked (i) whether the associated single nucleotide polymorphisms in this gene correlated with either clinical lung disease (ppFEV1) or nutritional state (BMI): they correlated only with BMI; (ii) whether the variants could be associated in any way with non‐*[F508del]*CFTR genotypes: they could not; and (iii) whether there were correlations with patient gender: there were none.

## 2 MATERIALS AND METHODS

### 2.1 Samples and phenotype data

Subjects (*n*=98) were recruited randomly from the UC San Diego adult CF clinic and had a clinical diagnosis of CF confirmed by either sweat chloride or CFTR genetic testing. CFTR genetic testing was obtained from CLIA‐certified commercial laboratories. All patients signed informed consent (UCSD Human Research Protection Program application #160078). All clinical data used for this study was obtained in the course of the subjects ongoing clinical care. In addition to the blood samples used for sequencing, the clinical data used in these studies included spirometry, nutritional, and clinical microbiology data.

### 2.2 Whole genome sequencing

Whole genome sequencing of the 98 CF patients was performed on the HiSeq X platform. The processing and analysis of whole genome sequencing data were performed using The American Genome Center (TAGC) pipelines. Briefly, BCL files were converted to FASTQ files by bcl2fastq software. 150 bp paired‐end reads were aligned to hg19 human reference genome using Isaac Aligner to generate BAM files. Using the resulting BAM files, single sample variant calls were made by Illumina Starling2 Variant Caller.

### 2.3 Sequencing quality assessment

Sequencing quality control was performed by Illumina Hiseq Analysis Software and Picard package. Quality assessment metrics used in the process include mean coverage depth, total number of uniquely aligned reads, percent alignment, and mean base quality. The WGS mean coverage was ~40×. To evaluate within‐sample contamination, we used the GATK ContEst program from the Broad Institute. We set ContEst 5% as the cutoff and excluded samples above that threshold from further analysis. Sex checks were also conducted using the ratio of the number of heterozygous SNPs over the number of homozygous SNPs on chromosome X, excluding pseudo autosomal regions (chrX:1–2,700,000 and chrX:154,000,000–155,270,560). Expected values are close to zero for males and around 1–1.5 for females.

### 2.4 Cohort VCF generation and annotation

To join variants from all samples, we used gvcfgenotyper (https://github.com/illumina/gvcfgenotyper) to merge per sample genome VCF files into a cohort VCF file. Multi‐allelic variants in the resulting cohort VCF file were split by bcftools into separate sites (https://github.com/samtools/bcftools). Cohort level variants on autosomal chromosomes (chromosome 1–22) and chromosome X were further filtered by the following criteria: (1) the proportion of samples with non‐reference alleles having a PASS filter (VQ, under FT tag) from individual sample genome VCF files is greater than 90%; and (2) The proportion of samples having a minimum genotype quality score of 20 (GQ ≥ 20) is greater than 90%. To provide functional annotation, the filtered cohort VCF was annotated by ANNOVAR program (Wang et al., 2010).

### 2.5 Manual validation of CFTR variants using IGV

All CFTR variants were manually validated with Integrative Genomics Viewer (IGV) and compared with the previously reported variant calls (Robinson et al., 2011; Thorvaldsdottir et al., 2013).

### 2.6 Statistical analysis

We used RVTESTS (version 20190205) for our association study (Zhan et al., 2016). We selected a single variant score test (score) to determine likelihood of gene associations between the homozygous and heterozygous [*F508del*]CFTR cohort. The covariates for this study were the first ten principal components from our principal component analysis (PCA) based on our cohort VCF. Additionally, we set the parameters of our score test to include only non‐silent exonic variants with a minor allele frequencies (MAF) based on the cohort (*n*=87) greater than 5%.

We utilized R(2.3.2, https://www.r‐project.org) for any additional statistical analysis. We used the SNPRelate package (1.4.2, http://bioconductor.org/packages/SNPRelate) to perform linkage disequilibrium (LD) and PCA on our cohort VCF file (Zheng et al., 2012). The false discovery rates (FDR) for our association data were calculated using the Benjamini & Hochberg (BH) method specified in the p.adjust function. We used robust linear regression (0.92–8, http://CRAN.R‐project.org/package=robustbase) to identify the significance between patient phenotype and TNS1 genotype with sex, age, and ancestry as covariates (Maechler et al., 2017).

## 3 RESULTS

### 3.1 Clinical Phenotyping and quality control for individual CFTR variants

**FIGURE 1:**
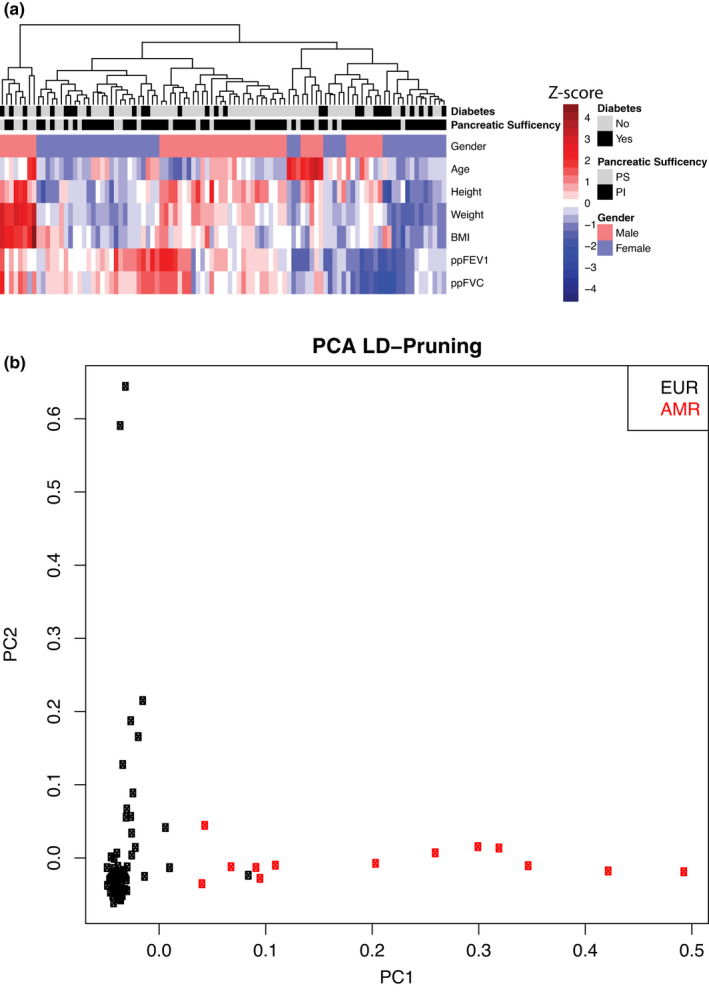
Population structure of the set of Cystic Fibrosis subjects available for analysis. (a) Hierarchical clustering of patient phenotype (scaled *Z*‐score). Data indicate a strong dependence on gender and lung function phenotypes. There are also areas where lung function does not correlate with nutritional indicators. (b) Principal component analysis showing patient differentiation based on LD pruning of 30% threshold and 750 k base pairs. This is the part of the strategy for simplifying the population

The patient cohort's characteristics are typical of an adult CF patient population. Table S1 shows basic demographic, nutritional, physiological, and clinical laboratory characteristics of the study population. Figure 1a shows a hierarchical clustering of clinical phenotypic information for 98 adult CF patients. Lung function was determined by using the best percent predicted FEV1% (ppFEV1) value within the last 6 months of measurement. The median BMI for repeated patient visits with varying degrees of frequency was calculated to minimize outliers. Elevated BMIs were not uniformly distributed (Shapiro–Wilk: *p* = 8.85E‐07). In addition, patient clustering appeared heavily dependent on lung function. High lung function and high BMIs were also observed regardless of diabetes and pancreatic status.

**FIGURE 2:**
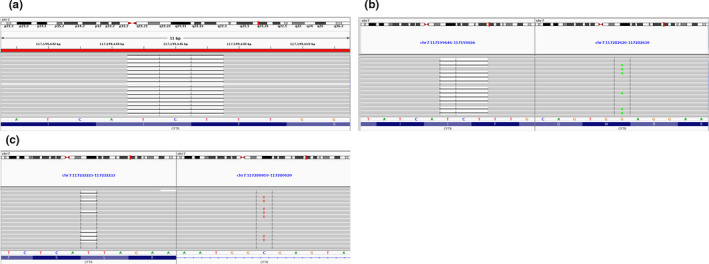
Images of variant calls for CF Patients. (a) Patient #108 is homozygous for [*F508del*]CFTR. (b) Patient #38 is compound heterozygous [*F508del*]CFTR. (c) Patient #76 is non‐[*F508del*]CFTR

As part of the preliminary quality control on patient identity, Figure 1b shows a Principal Component analysis for ancestry. The data show that 92% of subjects are of European origin. The remainder is 8% Ad Mixed American (AMR). Based on the variant calls of all the sequenced samples, we first determined CFTR variants for each patient by WGS (Table S2). Figure 2 shows examples of CF patients who are homozygous [*F508del*]CFTR, compound heterozygous [*F508del*]CFTR, and non‐[*F508del*]CFTR. WGS confirmed the Sanger‐based CFTR variant status for ninety‐eight out of ninety‐eight samples. In total, our final sequenced cohort consisted of 45 homozygous [*F508del*]CFTR, 42 compound heterozygous [*F508del*]CFTR, and 11 non‐[*F508del*]CFTR CF patients. However, in order to enable the search for a modifier gene that may have functional consequences for homozygous or compound heterozygous *[F508del]*CFTR patients, we removed the 11 non‐[*F508del*]CFTR CF patients. This strategy resulted in 87 high quality subjects for the analysis.

### 3.2 Experimental design emphasizes detection of non‐silent exonic variants

To account for ancestry effects confounding our results between the remaining 87 subjects in our CF cohort, we used the first ten principal components as genetic covariates in association testing. Principal Component analysis is a way to find the underlying structure of the dataset by dimensional reduction and maximizing variance. We combined principal component analysis with linkage disequilibrium (LD)‐based pruning by setting the threshold to 30% and 750 k base pairs. This strategy allowed us to avoid the strong influence of single nucleotide polymorphism (SNP) clustering while improving the differentiation between subjects within the cohort. We stopped at PC10 because of progressively smaller eigenvalues or dimensional insights into the structure of our data (Figure S1).

Additionally, we restricted our analysis to genetic variants that resulted in non‐silent protein‐coding effects because they may have a modifying effect on phenotype and because our cohort was of modest size. Thus while conventional CF association studies involve variations in patient phenotypes to identify genes associated with a particular trait, this new strategy allowed us to look for associations between homozygous and compound heterozygous *[F508del]*CFTR genotypes. The result was the possibility of identifying potential modifier genes that could not normally be detected with conventional CF trait associations.

### 3.3 A single variant score test identifies CFTR and Tensin 1 (*TNS1*)

**FIGURE 3:**
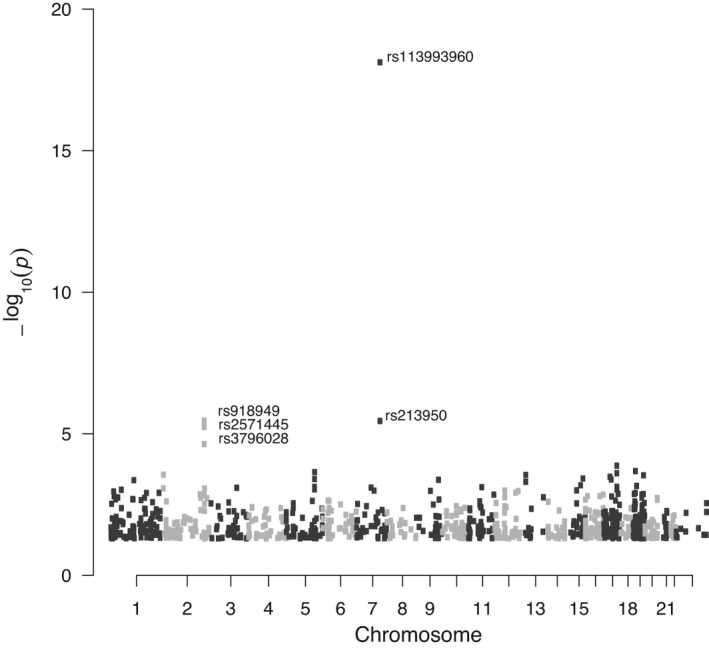
Manhattan plot showing genome wide variants

**FIGURE 4:**
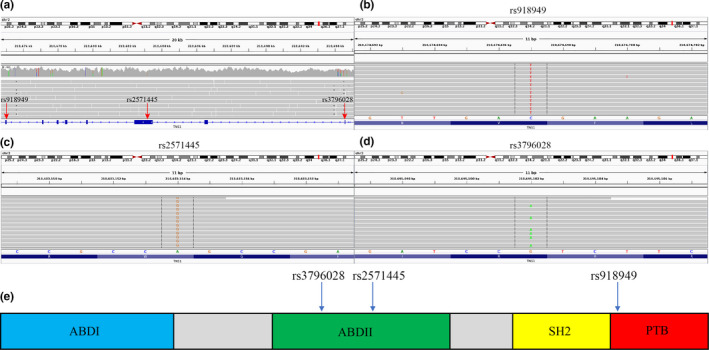
(a) Overview of TNS1 variant locations for patient #160 (b) TNS1 rs918949 C>T, (c) TNS1 rs2571445 A>G, (d) TNS1 rs3796028 G>A (e) Cartoon structure of TNS1. TNS1 918949 is located on the phosphotyrosine binding domain (PTB) which binds to the β1 tail of the fibronectin‐binding integrin, α5β1 integrin. TNS1 rs2571445 and TNS1 rs3796028 are located on the actin binding domain II (ABDII) region, which controls the polymerization of cytosolic actin

Table 1 and Table S3 summarize the results of a single variant score test, in which we tested for common variants (*n* = 17,219), that is MAF >5%, which distinguish between homozygous and compound heterozygous [*F508del*]CFTR genotypes. As shown in these data, the allele status of 4 SNPs were found to be significantly associated by CFTR variant status (FDR <0.1). As expected, [*F508del*]CFTR (rs113993960) was strongly associated with CFTR variant status (FDR <0.05). We also detected the CFTR variant *[M470V]*CFTR, frequently considered for its potential contribution to pathogenicity (Çelik et al., 2017; Ni et al., 2012; Nikolic et al., 2006). Additionally, we identified 3 non‐silent variants in the Tensin 1 gene (*TNS1)*, including rs918949, rs2571445, and rs3796028. Variants in Tensin 1 (*TNS1*), a gene not previously identified as a modifier gene for CF, have been associated with lung function (Hancock et al., 2010), but not with CFTR variant status. Figure 3 shows the Manhattan plot of the association results. The lowest *p* value is associated with [*F508del*]CFTR on chromosome 7. The *TNS1* variants have higher but still significant *p* values, and are located on chromosome 2. The locations of the TNS1 variants on the TNS1 gene are shown in Figure 4. *TNS1* (rs918949) is located on the phosphotyrosine binding domain (PTB) which binds to the β1 tail of the fibronectin‐binding integrin, α5β1 integrin. *TNS1* (rs2571445) and *TNS1* (rs3796028) are located on the actin binding domain II (ABDII) region, which controls the polymerization of cytosolic actin.

**TABLE 1:** Top 5 ranked association results from single variant score test

Gene	CHROM	POS	ID	REF	ALT	*p* Value	FDR
CFTR	chr7	117199644	rs113993960	ATCT	A	2.18E−19	3.75E−15
TNS1	chr2	218674697	rs918949	C	T	4.06E−06	3.50E−02
TNS1	chr2	218683154	rs2571445	A	G	7.10E−06	4.08E−02
CFTR	chr7	117199533	rs213950	G	A	1.58E−05	6.80E−02
TNS1	chr2	218695102	rs3796028	G	A	8.12E−05	2.80E−01

### 3.4 Variants in *TNS1* (rs918949) are associated with lower BMI for homozygous [*F508del*]CFTR patients

**FIGURE 5:**
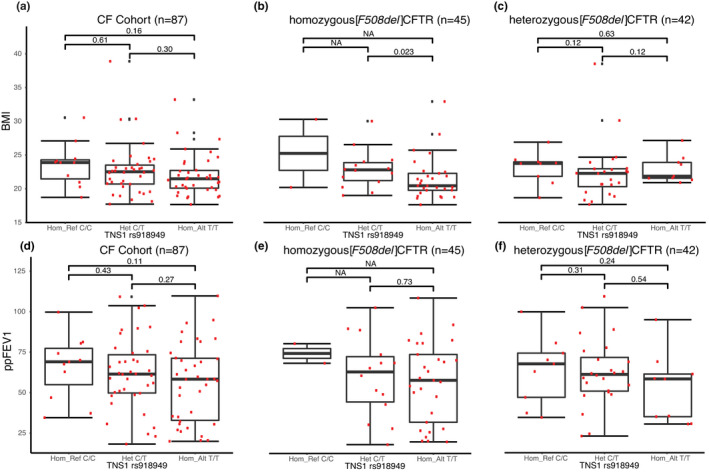
(a) Cohort BMI versus TNS1 rs918949 genotype (b) homozygous [*F508del*]CFTR BMI versus TNS1 rs918949 genotype (c) compound heterozygous [*F508del*]CFTR BMI versus TNS1 rs918949 genotype (d) Cohort ppFEV1 versus TNS1 rs918949 genotype (e) homozygous [*F508del*]CFTR ppFEV1 versus TNS1 rs918949 genotype (f) compound heterozygous [*F508del*]CFTR ppFEV1 versus TNS1 rs918949 genotype

To determine possible clinical features that further characterize the *TNS1* genotype vs CF genotype association, we proceeded to test phenotype groups. Based on available clinical phenotypes, we selected 6: ppFEV1, ppFVC, BMI, CF‐related diabetes, pancreatic sufficiency, and sex (Table S4). Of these we found one to be associated with TNS1 genotype. A comparison of the TNS1 (rs918949) genotype with the entire patient cohort BMI showed no statistically significant relationship (Figure 5a). However, Figure 5b shows that in the case of the *TNS1* (rs918949) genotype, homozygous [*F508del*]CFTR patients, who were also homozygous for rs918949*T*/*T*, had a significantly lower BMI than those who were heterozygous *C*/*T* (*p* = 0.023). However, the homozygous *C*/*C* genotype could not be analyzed in homozygous *[F508del]*CFTR patients because only two patients possessed it. In addition, because sex can be related to BMI, we then added sex to the analysis. An increase in BMI was detected in both sexes in the heterozygous individuals (females C/T 22.3 versus C/C 20.4, and males C/T 24.0 versus C/C 23.2). Females continue to be significant, but within males this analysis was not significant, potentially explained by the lower number of males in the analysis. By contrast, Figure 5c shows that in the case of CF patients heterozygous for *[F508del]*CFTR, the TNS1 rs918949*T*/*T* genotype was not associated with a lower BMI. This indicates that the association of TNS1 rs918949 with lower BMI is dependent on also having a homozygous [*F508del*]CFTR genotype (interaction *p* = 0.008). Finally, as shown in Figure 5d–f, no significant relationship could be detected between *TNS1* (rs918949) genotypes and pulmonary function (*viz*., ppFEV1). We interpreted this difference between dependence on BMI but not ppFEV1 to constitute a compelling control for the apparently specific relationship of the TNS1 variant rs918949 to BMI in homozygous [*F508del*]CFTR patients.

### 3.5 Variants in *TNS1* (rs2571445) are associated with lower BMI for homozygous [*F508del]CFTR* patients

**FIGURE 6:**
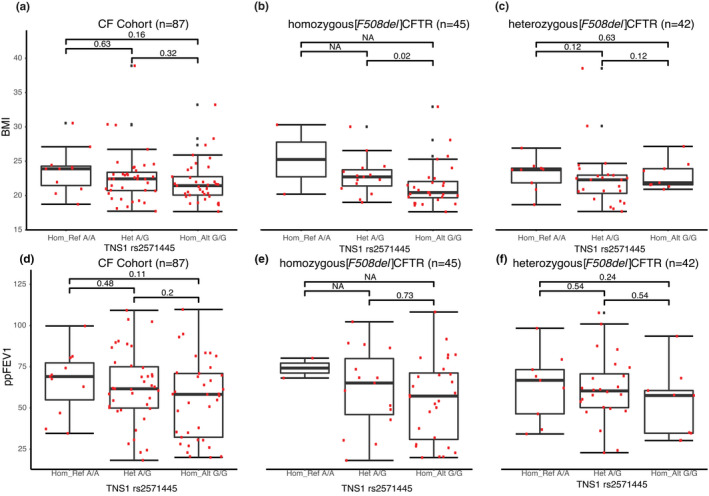
(a) Cohort BMI versus TNS1 rs2571445 genotype (b) homozygous [*F508del*]CFTR BMI versus TNS1 rs2571445 genotype (c) compound heterozygous [*F508del*]CFTR BMI versus TNS1 rs2571445 genotype (d) Cohort ppFEV1 versus TNS1 rs2571445 genotype (e) homozygous [*F508del*]CFTR ppFEV1 versus TNS1 rs2571445 genotype (F) compound heterozygous [*F508del*]CFTR ppFEV1 versus TNS1 rs2571445 genotype

A comparison of the TNS1 (rs2571445) genotype with the entire patient cohort BMI revealed no statistical difference with patient BMI (Figure 6a). However, Figure 6b shows that in the case of CF patients homozygous for [*F508del*]CFTR, and who were also homozygous for *TNS1* (rs2571445) *G*/*G*, there was a significantly lower BMI than for those patients who were heterozygous for *TNS1* (rs2571445) *A*/*G* (*p* = 0.02). Again, the homozygous *A*/*A* genotype could not be analyzed in homozygous *[F508del]*CFTR patients because only two patients possessed it. Furthermore, because sex can be related to BMI, we again added sex to the analysis. An increase in BMI was detected in both sexes in the heterozygous individuals (females A/G 22.3 vs G/G 20.4, and males A/G 24.0 vs G/G 23.2). Females continue to be significant, but within males this analysis was once again not significant, potentially explained by the lower number of males in the analysis. Figure 6c also shows that in the case of CF patients who were compound heterozygous *[F508del]CFTR*, the *TNS1* rs2571445 *A*/*A* genotype was not associated with a lower BMI. This indicates that the modifier gene status of the *TNS1* rs2571445 variant is dependent on a homozygous [*F508del*]CFTR genotype (interaction *p*‐value = 0.005). Finally, as shown in Figure 6d–f, no significant relationship could be detected between *TNS1* (rs2571445) genotypes and pulmonary function (*viz*, ppFEV1).

### 3.6 Distribution of *TNS1* (rs918949) and (rs2571445) genotypes in patients with at least one copy of [*F508del*]CFTR

Table 2 shows that homozygous [*F508del*]CFTR patients were associated with a plurality of the TNS1 rs918949T/T and rs2571445 G/G genotypes. By comparison, compound heterozygous *[F508del]*CFTR patients consisted mostly of the heterozygous *TNS1* genotypes. Furthermore, we found that the similarities between *TNS1* (rs918949), *TNS1* (rs2571445), and the BMI results were due to patients having the same genotypes for both SNPs. Finally, the linkage disequilibrium analysis confirmed that rs918949(C) allele was correlated with rs2571445(A) allele, and the rs918949(T) allele was correlated with rs2571445(G) allele (*D*ʹ = 0.96, *R* ^2^ = 0.88). Thus the data indicate that a patient who had *TNS1* (rs918949) T/T would also have rs2571445 (G/G). These results indicate that having just one of the TNS1 variants alone may not have a modifying effect; or that there is a possible cooperative effect of the *TNS1* variants; or that there is a survival advantage to having both variants for the CF patient.

**TABLE 2:** Distribution of TNS1 variants in homozygous and compound heterozygous [*F508del*]CFTR patients

	TNS1 rs918949 (C>T)	TNS1 rs2571445 (A>G)	TNS1 rs3796028 (G>A)
Homozygous [*F508del*]CFTR			
Reference	2	2	26
Heterozygous	14	15	19
Homozygous	29	28	0
Heterozygous [*F508del*]CFTR			
Reference	9	9	11
Heterozygous	24	24	25
Homozygous	9	9	6

### 3.7 Variants in *TNS1 (*rs3796028) have no association with BMI or ppFEV1

*TNS1* (rs3796028) is the variant with the highest *p*‐value. However, when we compared BMI and ppFEV1 with this variant we observed no significant difference between the *TNS1* genotype regardless of patient CFTR variant. Retrospectively, this could also be due to too few patients being homozygous for the *TNS1* (rs3796028 A/A).

## 4 DISCUSSION

In this paper, we have used whole genome sequencing to identify the *TNS1* gene as a potential modifier gene for BMI in CF patients who are either homozygous or compound heterozygous for [*F508del*]CFTR. The homozygous *[F508del]*CFTR patients had 3 times the number of homozygous rs918949 and rs2571445 *TNS1* variants than compound heterozygous [*F508del*]CFTR patients (Table 2). However, while *TNS1* has not been historically associated with any CF phenotype, it has been associated with COPD and asthma lung function (Ferreira et al., 2014; Soler et al., 2011). Association studies on CF phenotypes ranging from lung function to meconium ileus have resulted in identification of various potential modifier genes for CF (Corvol et al., 2015; Di Paola et al., 2017; Gong et al., 2019). However, to our knowledge none have yet identified modifier genes associated with BMI. Furthermore, to this end, none have utilized whole genome sequencing. Additionally, we are unaware of association studies performed based specifically on CF patient genotypes. This approach may therefore provide insight into potential modifier genes not normally seen in trait comparisons. Importantly, these data thus confirm the statistical value of limiting variation to those exonic sequences bearing non‐silent variants. In retrospect, the consequences of this global strategy did not prevent us from finding [*M470V*], a previously known candidate modifier variant, within the CFTR gene itself. Thus this epistatic strategy has both microscopic and macroscopic capabilities for modifier gene discovery. Based on these aggregate data, we conclude that these newly identified variants in *TNS1* may be modifiers for homozygous [*F508del*]CFTR CF patients who have BMIs that indicate an overweight or approaching overweight status.

It is possible that a relationship between *TNS1* and BMI might be interpretable on the basis of what is known about *TNS1* function. For example, as shown in Figure 4, Tensin 1 is a multidomain protein that consists of two actin binding domains (ABDI & ABDII), a Src homology (SH2), and a phosphotyrosine‐binding (PTB) domain (Lo, 2004). The ABDI domain is known to interact with the actin cytoskeleton, and the ABDII domain retards the actin filament polymerization. The SH2 domain is known to interact with focal adhesion‐like kinases, such as focal adhesion kinase (FAK), p130 Crk‐associated substrate, and phosphoinositide 3‐kinase. The PTB domain was originally thought to interact with tyrosine‐phosphorylated proteins, but studies have shown that it binds to the β1 tail of the α5β1 integrin. Thus the *TNS1* SNPs of interest are located on the ABDII (rs2571445), which interacts with cytosolic actin, and on PTB region (rs918949) that interacts with an integrins in the extracellular matrix. In sum, these *TNS1* variants may affect the bridge between cytosol and the extracellular matrix, with functional consequences for the BMI.

To understand how the functional connection between TNS1 variants and BMI could be manifested, we considered the fact that Tensin 1 has been shown to drive the assembly of fibronectin by binding to the β1 unit of the principal fibronectin‐binding integrin α5β1. Tensin 1 then moves the integrin from the cell periphery centripetally along the cell membrane. During this process the α5β1 integrin connects fibronectin fibrils, thereby reconstructing the ECM. Once localized, the tensin‐bound α5β1 can undergo endocytosis through an Arf4‐dependent pathway. It turns out that this process is inhibited by the nutrient‐sensing kinase mTORC1 during states of nutrient abundance. However, during a period of nutrient scarceness AMP‐kinase is upregulated and phosphorylates the Raptor component of mTORC1 to deactivate mTORC1. This process allows for the uptake of the tensin‐bound integrin and scavenging of the ECM during metabolic stress (Dornier & Norman, 2017; Georgiadou et al., 2017). We speculate, therefore, that these variants could engender CF patients with steadily normal or higher BMIs. For example, scavenging the ECM for sugars and amino acids could be one pathway that tries to correct for the energy loss via CF related fat malabsorption or pancreatic insufficiency. Since fat malabsorption in CF is chronic, the scavenging of the ECM may also help maintain the weight of the CF patient.

**FIGURE 7:**
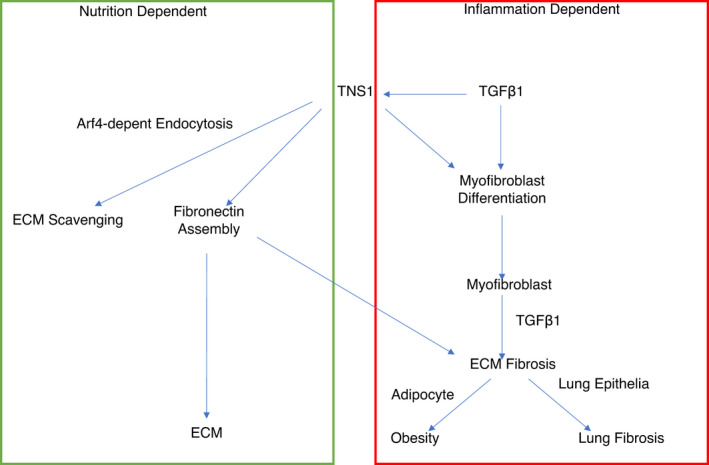
TNS1 plays a role in fibronectin assembly in the ECM and ECM scavenging (GREEN BOX), Inflammation via TGFB1 increases the expression of TNS1 leading to cell differentiation into myofibroblasts and upregulates TNS1 in myofibroblasts to increase ECM component production, such as collagen and fibronectin which leads to fibrosis. (RED BOX)

Figure 7 shows that although the aforementioned mechanism is nutrition dependent, chronic inflammation may also contribute to the difference in patient BMIs. For example, studies have shown that obesity and insulin resistance can be caused by inflammation and fibrosis related alterations to the ECM of fatty tissue (Chun, 2012; Lin et al., 2016; Sun et al., 2013). This occurs as a result of a more rigid ECM structure forming and preventing normal adipogenesis by hindering outward growth of adipocytes. This process could lead to adipose tissue dysfunction and subsequent obesity. Therefore, CF patients suffering from chronic inflammation and increased TNS1 expression may have a greater degree of adipose tissue dysfunction.

In addition, as summarized in detail in Figure S2, it is known that inflammation via *TGFB1* can cause multiple cell types to differentiate into myofibroblasts (Caam et al., 2018). Recently it has been shown that TGFB1 increases TNS1 expression to induce fibroblast cell differentiation into wound healing myofibroblasts (Bernau et al., 2017). Not only is TNS1 required for this differentiation process, but it is also necessary for myofibroblasts to produce the ECM components, including collagen I, fibronectin, and fibronectin containing extra type III domain A (EDA) (Bernau et al., 2017). Therefore, TNS1 is integral to fibrosis and the overall wound healing response.

Finally, our study has a number of limitations. First, all patient data was collected from a single pulmonary center (UCSD). This effectively keeps treatment and environment as a constant, yet provides only a small number of patients when compared to association studies utilizing data from multiple clinical centers. Secondly, our patient numbers limited our sub‐ grouping to those homozygous or compound heterozygous for the [*F508del*]CFTR variant, which may mask the effect of *TNS1* on the heterozygous sub‐group. Lastly, our cohort data had limited individuals who were homozygous for rs918949 C/C and rs2571445 G/G reference alleles, which prevented us from determining trend behaviors, or significant differences between those patients who were homozygous for the variant allele and homozygous reference allele.

In conclusion, we have observed significantly lower BMIs in homozygous [*F508del*]CFTR patients who are also homozygous for Tensin 1 variants rs918949 (T/T) and rs2571445 (G/G). Tensin 1 was the only potential modifier gene to be associated with CF sub‐groups. Finally, Tensin 1 showed no statistical difference with respect to patient lung function even though it has been previously associated with other lung diseases. Thus the Tensin 1 gene could be a modifier gene for low BMI in CF patients with the homozygous [*F508del*]CFTR variant.

## CONFLICT OF INTEREST

Authors declare they have no competing interests.

## AUTHOR CONTRIBUTIONS

H.B.P, D.C, N.I.W, and M.D.W conceptualized and designed the experiment. D.C collected samples and clinical data. C.L.D contributed to receiving, handling, and performing whole genome sequencing on samples. X.Z and M.D.W contributed to alignment and quality control of whole genome sequencing data. J.S, N.I.W, X.Z, and A.R.S contributed to data interpretation, statistical analysis, and visualization. H.B.P and M.D.W supervised the research and analysis. N.I.W and H.B.P drafted the manuscript. All authors contributed to reading, editing, and approving the final draft.

## Supporting information



Figure S1

Figure S2

Table S1

Table S2

Table S3

Table S4
