# Supplementary figures and images for: Tensin 1 (TNS1) is a modifier gene for low body mass index (BMI) in homozygous [F508del]CFTR patients
Source: Physiol Rep. 2021 Jun 4;9(11):e14886. doi: 10.14814/phy2.14886 (PMC8176904; doi:10.14814/phy2.14886)

Figure S11: Variance explained by PC1 to PC10

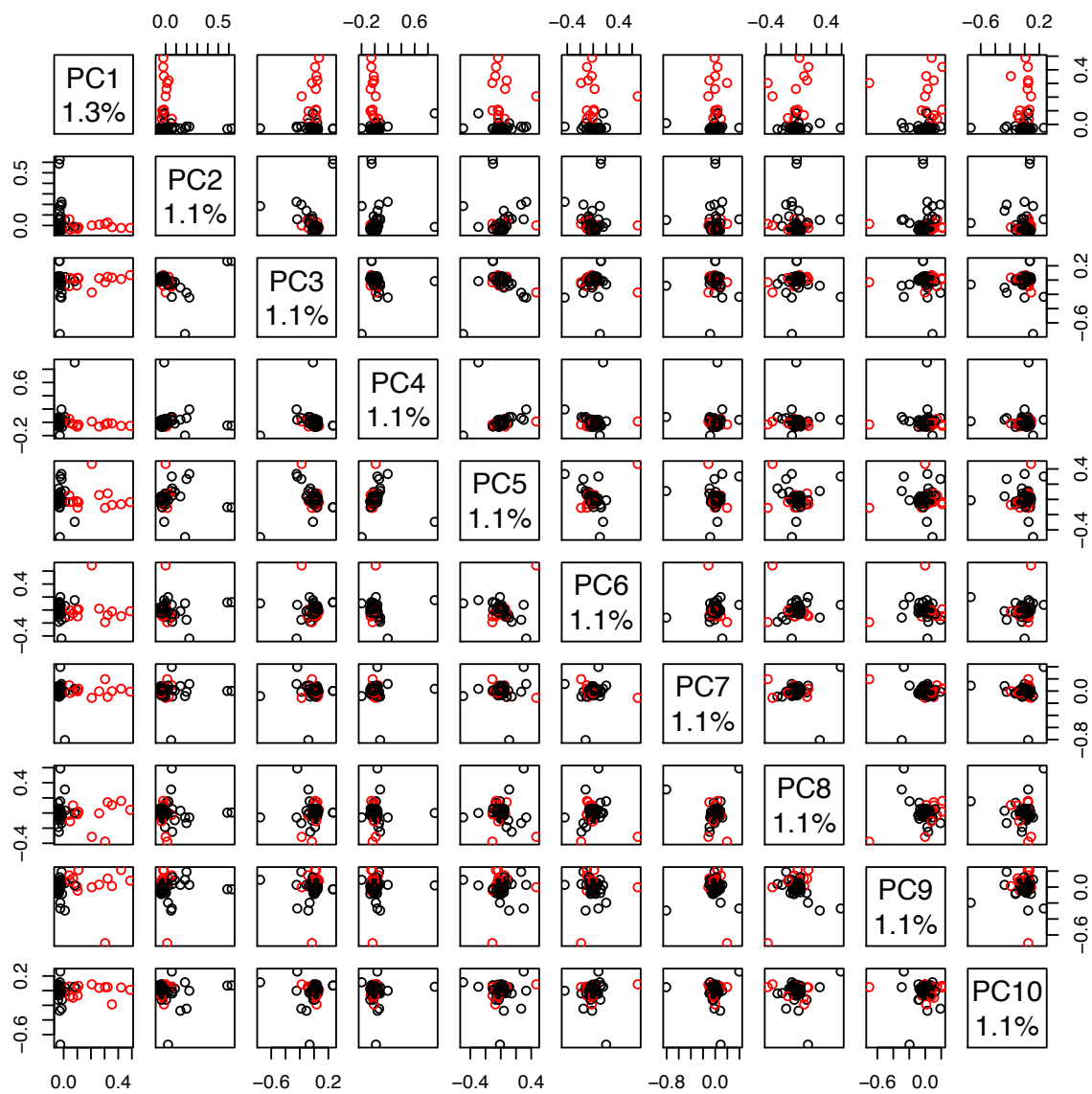

Supplement: Supplementary file 1 — Figure S1 [file PHY2-9-e14886-s003.pdf]
