# Supplementary material for: Tensin 1 (TNS1) is a modifier gene for low body mass index (BMI) in homozygous [F508del]CFTR patients
Source: Physiol Rep. 2021 Jun 4;9(11):e14886. doi: 10.14814/phy2.14886 (PMC8176904; doi:10.14814/phy2.14886)
Supplement: Supplementary file 2 — Figure S2 [file PHY2-9-e14886-s006.pdf]

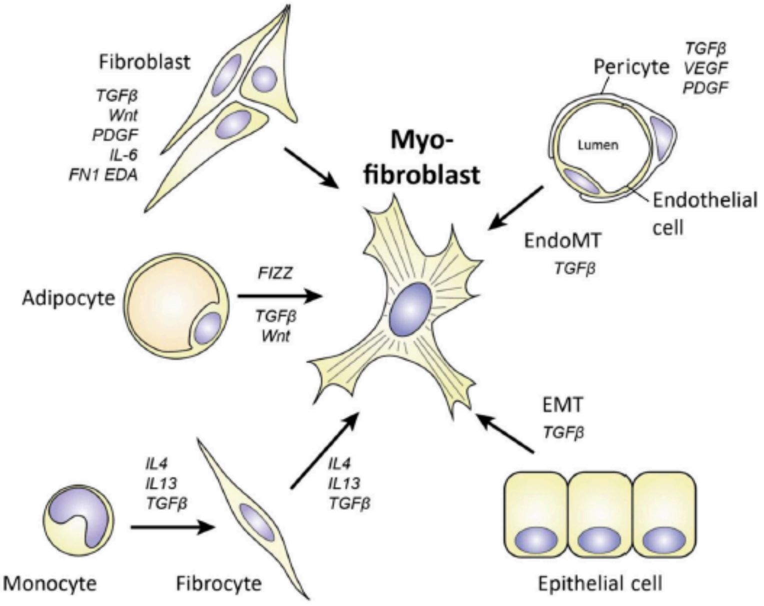

Figure SI1: Cellular origins of myofibroblasts in SSc. Myofibroblasts can originate from various cell types, including fibroblast, adipocytes, monocytes/fibrocytes, pericytes, endothelial cells, and epithelial cells. Key molecules for each transition are depicted. For epithelial cells to become myofibroblasts, they have to undergo epithelial to mesenchymal transition (EMT). For endothelial cells a similar process is needed, called endothelial to mesenchymal transition (EndoMT). **van Caam A, Vonk M, van den Hoogen F, van Lent P, van der Kraan P. Unraveling SSc Pathophysiology; The Myofibroblast. Front Immunol. 2018;9:2452.**
