# Supplementary material for: Tensin 1 (TNS1) is a modifier gene for low body mass index (BMI) in homozygous [F508del]CFTR patients
Source: Physiol Rep. 2021 Jun 4;9(11):e14886. doi: 10.14814/phy2.14886 (PMC8176904; doi:10.14814/phy2.14886)
Supplement: Supplementary file 3 — Table S1 [file PHY2-9-e14886-s001.pdf]

Table SI1: demographic, nutritional, physiological and clinical laboratory characteristics of the study population.

	All	homozygous *[delF508]*CFTR	compound heterozygous *[delF508]*CFTR	non-*[delF508]*CFTR
n	98	45	42	11
Male (%)	50.0	46.7	54.8	45.5
Age	35.9 (19-74)	35.8 (19-61)	36.2 (19-74)	35.1 (21-71)
EUR/AMR	85/13	42/3	33/9	10/2
ppFEV1	60.9 (18.2-112.3)	58.9 (18.2-109.7)	60.6 (23.0-109.2)	70.2 (30.2-112.3)
ppFVC	78.6 (32.4-122.0)	78.1 (32.4-122.0)	77.2 (37.9-110.7)	85.9 (45.3-111.7)
Height	168.8 (139.7-191.0)	170.0 (139.7-191.0)	167.6 (154.9-188.0)	168.7 (149.9-191.0)
Weight	60.8 (44.2-113.3)	61.0 (44.2-109.75)	60.8 (45.0-113.3)	59.4 (45.5-106.7)
BMI	22.6 (16.3-38.9)	22.2 (17.7-33.2)	22.8 (17.7-38.9)	23.4 (16.3-29.1)
Pancreatic Insufficient (%)	76.5	91.1	71.4	36.4
Diabetes (%)	30.6	44.4	16.7	27.3
PA (%)	71.1	73.3	69.0	72.7
MSRA (%)	20.4	24.4	16.7	18.2
MSSA (%)	29.6	26.7	28.6	45.5
