# Supplementary material for: Tensin 1 (TNS1) is a modifier gene for low body mass index (BMI) in homozygous [F508del]CFTR patients
Source: Physiol Rep. 2021 Jun 4;9(11):e14886. doi: 10.14814/phy2.14886 (PMC8176904; doi:10.14814/phy2.14886)
Supplement: Supplementary file 4 — Table S2 [file PHY2-9-e14886-s002.pdf]

Table SI2: CFTR and TNS1 variant calls for patient cohort.

PatientID	Gender	CFTR1	CFTR2	group	TNS1 (rs918949)	TNS1 (rs2571445)	TNS1 (rs3796028)
12	Female	F508del	F508del	1	0/1	0/1	0/1
69	Male	F508del	F508del	1	0/1	0/1	0/1
82	Male	F508del	F508del	1	0/1	0/1	0/1
160	Male	F508del	F508del	1	1/1	1/1	0/1
167	Female	F508del	F508del	1	1/1	1/1	0/1
176	Male	F508del	F508del	1	0/1	0/1	0/1
275	Female	F508del	F508del	1	1/1	1/1	0/1
282	Female	F508del	F508del	1	1/1	1/1	0/1
301	Female	F508del	F508del	1	0/1	0/1	0/1
325	Female	F508del	F508del	1	1/1	1/1	0/1
353	Female	F508del	F508del	1	0/1	0/1	0/1
371	Male	F508del	F508del	1	0/1	0/1	0/1
374	Male	F508del	F508del	1	0/1	0/1	0/1
377	Male	F508del	F508del	1	1/1	1/1	0/1
383	Female	F508del	F508del	1	0/1	0/1	0/1
385	Male	F508del	F508del	1	0/1	0/1	0/1
375	Male	F508del	F508del	1	1/1	0/1	0/1
86	Male	F508del	F508del	1	0/0	0/0	0/1
74	Male	F508del	F508del	1	0/0	0/0	0/1
10	Male	F508del	F508del	1	1/1	1/1	0/0
151	Female	F508del	F508del	1	1/1	1/1	0/0
35	Male	F508del	F508del	1	1/1	1/1	0/0
41	Male	F508del	F508del	1	1/1	1/1	0/0
42	Female	F508del	F508del	1	1/1	1/1	0/0
53	Male	F508del	F508del	1	1/1	1/1	0/0
141	Female	F508del	F508del	1	0/1	0/1	0/0
193	Female	F508del	F508del	1	1/1	1/1	0/0
147	Female	F508del	F508del	1	1/1	1/1	0/0
228	Female	F508del	F508del	1	1/1	1/1	0/0
92	Male	F508del	F508del	1	1/1	1/1	0/0
107	Male	F508del	F508del	1	1/1	1/1	0/0
108	Female	F508del	F508del	1	1/1	1/1	0/0
112	Male	F508del	F508del	1	1/1	1/1	0/0
243	Male	F508del	F508del	1	1/1	1/1	0/0
247	Female	F508del	F508del	1	0/1	0/1	0/0
251	Female	F508del	F508del	1	0/1	0/1	0/0
252	Female	F508del	F508del	1	0/1	0/1	0/0

200	Male	F508del	F508del	1	1/1	1/1	0/0
285	Female	F508del	F508del	1	1/1	1/1	0/0
290	Female	F508del	F508del	1	1/1	1/1	0/0
229	Female	F508del	F508del	1	1/1	1/1	0/0
264	Male	F508del	F508del	1	1/1	1/1	0/0
356	Female	F508del	F508del	1	1/1	1/1	0/0
265	Female	F508del	F508del	1	1/1	1/1	0/0
268	Female	F508del	F508del	1	1/1	1/1	0/0
379	Female	F508del	1312A->G	2	0/1	0/1	1/1
148	Female	F508del	1471delA	2	0/1	0/1	0/1
363	Male	F508del	1524+1delG	2	0/1	0/1	0/1
157	Female	F508del	1717-1G->A	2	0/1	0/1	0/1
184	Female	F508del	1717-1G->A	2	0/0	0/0	1/1
381	Male	F508del	1717-1G->A	2	0/0	0/0	0/0
104	Female	F508del	1898+1G->C	2	0/0	0/0	1/1
256	Male	F508del	1924del7	2	1/1	1/1	0/1
46	Female	F508del	2184insA	2	0/1	0/1	0/1
299	Female	F508del	2184insA	2	0/1	0/1	0/1
126	Female	F508del	2622+1G->A	2	0/1	0/1	0/1
234	Female	F508del	3120G->A	2	1/1	1/1	0/0
210	Male	F508del	3272-26A->G	2	0/1	0/1	0/1
239	Male	F508del	3849+10kbC->T	2	1/1	1/1	0/0
103	Female	F508del	3905insT	2	0/1	0/1	0/1
365	Male	F508del	3905insT	2	1/1	1/1	0/1
138	Male	F508del	3905insT	2	0/0	0/0	1/1
145	Female	F508del	621+1G->T	2	0/1	0/1	0/1
18	Female	F508del	621+1G->T	2	1/1	1/1	0/0
222	Female	F508del	A455E	2	1/1	1/1	0/0
140	Female	F508del	c.1210 -11T->G	2	0/0	0/0	0/1
278	Male	F508del	c.40delAA	2	1/1	1/1	0/0
272	Male	F508del	E257X	2	0/1	0/1	0/1
169	Male	F508del	G542X	2	1/1	1/1	0/1
225	Male	F508del	G542X	2	0/0	0/0	1/1
61	Female	F508del	G551D	2	0/1	0/1	0/1
269	Male	F508del	G551D	2	0/1	0/1	0/0
230	Male	F508del	L206W	2	0/0	0/0	1/1
177	Male	F508del	P67L	2	0/1	0/1	0/1
146	Male	F508del	Q1382X	2	1/1	1/1	0/0
66	Female	F508del	Q372Q	2	0/1	0/1	0/1
262	Female	F508del	R1158X	2	0/1	0/1	0/0

77	Male	F508del	R117H	2	0/1	0/1	0/1
288	Male	F508del	R117H	2	0/1	0/1	0/0
254	Male	F508del	R347H	2	0/1	0/1	0/1
180	Male	F508del	R352Q	2	0/1	0/1	0/1
260	Male	F508del	R560T	2	0/1	0/1	0/1
259	Female	F508del	T1246I	2	0/1	0/1	0/1
178	Male	F508del	W1282X	2	0/1	0/1	0/1
38	Male	F508del	W1282X	2	0/0	0/0	0/1
192	Female	F508del	W1282X	2	0/0	0/0	0/1
27	Male	F508del	W1282X	2	0/1	0/1	0/0
232	Female	G576A	-741T->G	3	1/1	1/1	0/0
76	Male	3849+10kbC->T	2143delT	3	0/1	0/1	0/1
114	Male	1078delT	2789+5G->A	3	1/1	1/1	0/0
302	Female	I507del	2896insAG	3	0/1	0/1	0/1
253	Female	Q98R	297-1G->A	3	0/1	0/1	0/1
338	Male	2184delA	3272-26A->G	3	1/1	1/1	0/0
1	Female	R117H	621+1G->T	3	1/1	1/1	0/0
237	Female	G551D	621+1G->T	3	0/1	0/1	0/0
328	Female	R117H	G551D	3	0/1	0/1	0/0
318	Male	W1282X	L206W	3	0/1	0/1	0/0
344	Male	G542X	R347P	3	0/1	0/1	0/1
