# Supplementary material for: Tensin 1 (TNS1) is a modifier gene for low body mass index (BMI) in homozygous [F508del]CFTR patients
Source: Physiol Rep. 2021 Jun 4;9(11):e14886. doi: 10.14814/phy2.14886 (PMC8176904; doi:10.14814/phy2.14886)
Supplement: Supplementary file 5 — Table S3 [file PHY2-9-e14886-s005.pdf]

Table SI3: Association Results with p-values less than or equal to 0.05

CHROM	POS	GENE	SNP	REF	ALT	AF	PVALUE	FDR
chr7	117199644	CFTR	rs113993960	ATCT	A	0.758621	7.56E-19	1.30E-14
chr2	218674697	TNS1	rs918949	C	T	0.655172	3.46E-06	0.02037369
chr7	117199533	CFTR	rs213950	G	A	0.91954	3.55E-06	0.02037369
chr2	218683154	TNS1	rs2571445	A	G	0.649425	5.80E-06	0.0249629
chr2	218695102	TNS1	rs3796028	G	A	0.321839	2.32E-05	0.07986
chr17	67125840	ABCA6	rs4968839	C	T	0.683908	0.00013489	0.38711182
chr19	12256912	ZNF625	rs7258368	C	T	0.166667	0.00020835	0.39710458
chr5	140626627	PCDHB15	rs618096	G	A	0.568966	0.00022649	0.39710458
chr17	67081830	ABCA6	rs2302134	T	C	0.643678	0.00024798	0.39710458
chr2	15607842	NBAS	rs4668909	T	C	0.66092	0.00028231	0.39710458
chr13	25487103	CENPJ	rs35498994	T	C	0.103448	0.00028729	0.39710458
chr19	52660279	ZNF836	rs1366245	C	T	0.201149	0.00029388	0.39710458
chr17	33433487	RAD51L3	rs4796033	C	T	0.178161	0.00033559	0.39710458
chr17	33433487	RAD51L3-RFFL	rs4796033	C	T	0.178161	0.00033559	0.39710458
chr15	90344352	ANPEP	rs8192297	T	C	0.166667	0.00038191	0.39710458
chr5	140573754	PCDHB10	rs702386	A	C	0.586207	0.00039872	0.39710458
chr17	39661366	KRT13	rs760134	G	C	0.0747126	0.00041548	0.39710458
chr9	112082510	EPB41L4B	rs117569740	C	T	0.16092	0.00042346	0.39710458
chr1	117311147	CD2	rs699738	C	A	0.12069	0.00043818	0.39710458
chr13	25428002	RNF17	rs3783082	C	A	0.114943	0.00049825	0.42896834
chr15	78882925	CHRNA5	rs16969968	G	A	0.344828	0.00066096	0.44785779
chr19	18272190	PIK3R2	rs2241088	A	C	0.87931	0.0006998	0.44785779
chr17	67178316	ABCA10	rs4968849	A	G	0.718391	0.0007513	0.44785779
chr5	140531746	PCDHB6	rs246703	C	G	0.597701	0.00075641	0.44785779
chr11	65810045	GAL3ST3	rs4565902	T	G	0.149425	0.00077116	0.44785779
chr5	140553581	PCDHB7	rs2910313	G	C	0.591954	0.00079079	0.44785779
chr7	73097654	DNAJC30	rs1128349	C	T	0.413793	0.00079846	0.44785779
chr3	138289221	CEP70	rs1673607	C	T	0.528736	0.00081621	0.44785779

chr2	15674686	NBAS	rs13029846	T	C	0.649425	0.00084983	0.44785779
chr2	220037756	CNPPD1	rs1043160	A	G	0.609195	0.00086378	0.44785779
chr2	220037666	CNPPD1	rs1127102	A	G	0.603448	0.0008818	0.44785779
chr5	140559914	PCDHB8	rs2740583	T	C	0.344828	0.00092724	0.44785779
chr17	67212423	ABCA10	rs9909216	G	A	0.649425	0.00093637	0.44785779
chr1	54605318	CDCP2	rs3841798	T	TG	0.183908	0.00095516	0.44785779
chr1	54605318	CDCP2	rs76608277	T	TG	0.183908	0.00095516	0.44785779
chr15	59499179	LDHAL6B	rs3809530	G	A	0.304598	0.00100301	0.44785779
chr15	59500116	LDHAL6B	rs3825937	T	C	0.304598	0.00100301	0.44785779
chr12	50189602	NCKAP5L	rs3813526	A	G	0.0632184	0.00102124	0.44785779
chr12	50190667	NCKAP5L	rs3741554	A	T	0.0632184	0.00102124	0.44785779
chr7	88424115	C7orf62	rs2373396	C	G	0.16092	0.00102222	0.44785779
chr9	71851877	TJP2	rs34774441	G	A	0.0689655	0.0010476	0.44785779
chr12	117175608	C12orf49	rs73220422	C	T	0.0574713	0.00106639	0.44785779
chr1	16389026	FAM131C	rs2863458	T	C	0.327586	0.00109854	0.45037524
chr12	104153004	STAB2	rs2271637	C	G	0.298851	0.00121591	0.45084837
chr1	242035438	EXO1	rs4149965	G	A	0.224138	0.00122388	0.45084837
chr1	179504025	AXDND1	rs141228272	AAAG	A	0.344828	0.00123058	0.45084837
chr1	179504043	AXDND1	rs6658180	C	G	0.344828	0.00123058	0.45084837
chr19	12222861	ZNF788	rs1975349	T	C	0.206897	0.00123061	0.45084837
chr17	73498483	CASKIN2	rs7503373	T	C	0.885057	0.00132707	0.45559242
chr19	6156483	ACSBG2	rs4807840	T	C	0.672414	0.00135098	0.45559242
chr19	52033206	SIGLEC6	rs2005199	G	A	0.0747126	0.00135747	0.45559242
chr2	197990741	ANKRD44	rs35338671	A	C	0.0632184	0.00139056	0.45559242
chr16	89167094	ACSF3	rs7188200	T	C	0.741379	0.00141273	0.45559242
chr19	43699417	PSG4	rs3859474	A	G	0.454023	0.00141525	0.45559242
chr11	126081403	RPUSD4	rs2282580	T	C	0.385057	0.00142877	0.45559242
chr2	218669225	TNS1	rs61741262	T	C	0.114943	0.00147255	0.46101524
chr16	11367154	PRM3	rs429744	C	T	0.942529	0.00160311	0.47028583
chr1	19186129	TAS1R2	rs9701796	G	C	0.735632	0.00160574	0.47028583
chr16	72991715	ZFHX3	rs4788682	A	G	0.833333	0.00161191	0.47028583

chr17	41246481	BRCA1	rs1799950	T	C	0.0689655	0.00164538	0.47028583
chr12	50232169	BCDIN3D	rs11169172	A	T	0.0804598	0.0017388	0.47028583
chr13	113053470	SPACA7	rs10816	T	A	0.649425	0.00175462	0.47028583
chr1	38185723	EPHA10	rs6670599	C	T	0.091954	0.00178442	0.47028583
chr1	38188740	EPHA10	rs12405650	C	T	0.091954	0.00178442	0.47028583
chr1	38188787	EPHA10	rs17511304	A	G	0.091954	0.00178442	0.47028583
chr19	43763144	PSG9	rs2355447	C	T	0.0747126	0.0017955	0.47028583
chr2	201354935	KCTD18	rs3795969	C	G	0.431034	0.00182225	0.47028583
chr1	170521376	GORAB	rs913257	G	A	0.396552	0.00183483	0.47028583
chr20	61512185	DIDO1	rs41282984	G	C	0.0689655	0.00186162	0.47028583
chr2	232457880	C2orf57	rs74460533	T	C	0.0574713	0.00188453	0.47028583
chr1	110766454	KCNC4	rs59123361	G	A	0.137931	0.0020379	0.48286381
chr1	27339103	FAM46B	rs4970471	G	T	0.183908	0.0020653	0.48286381
chr1	113098534	ST7L	rs6658555	C	T	0.252874	0.00209003	0.48286381
chr9	114462322	C9orf84	rs6477845	A	G	0.787356	0.00212269	0.48286381
chr9	114464487	C9orf84	rs1407390	T	C	0.787356	0.00212269	0.48286381
chr9	114490308	C9orf84	rs7470491	T	C	0.787356	0.00212269	0.48286381
chr16	840378	CHTF18	rs3765263	A	G	0.241379	0.00213123	0.48286381
chr20	62422080	ZBTB46	rs2281929	T	C	0.091954	0.00216343	0.48327802
chr1	152882610	IVL	rs2229496	A	G	0.126437	0.00221776	0.48327802
chr1	152883711	IVL	rs7545520	G	C	0.126437	0.00221776	0.48327802
chr17	60741917	MRC2	rs2014055	G	A	0.12069	0.00229243	0.48327802
chr6	27216699	PRSS16	rs5030965	G	T	0.0517241	0.00229374	0.48327802
chr6	32548632	HLA-DRB1	rs1136881	T	A	0.218391	0.00230972	0.48327802
chr5	129521126	CHSY3	rs2015018	A	G	0.735632	0.00236412	0.48327802
chr2	30975961	CAPN13	rs62142192	G	T	0.155172	0.00245862	0.48327802
chr11	56000403	OR5T2	rs10791893	C	G	0.91954	0.00246349	0.48327802
chr19	8808900	ACTL9	rs10410943	A	G	0.735632	0.0024811	0.48327802
chr19	8808942	ACTL9	rs2340550	A	G	0.735632	0.0024811	0.48327802
chr19	9236698	OR7G3	rs111279560	G	GATGGT	0.321839	0.00252577	0.48327802
chr19	9237542	OR7G3	rs10414255	T	C	0.321839	0.00252577	0.48327802

chr14	23744932	HOMEZ	rs1055061	C	T	0.0689655	0.00252599	0.48327802
chr1	209950760	TRAF3IP3	rs669694	C	G	0.793103	0.002613	0.49443129
chr3	97983265	OR5H6	rs4241472	C	A	0.0977011	0.00268509	0.50254962
chr12	7249598	C1RL	rs3742089	T	C	0.454023	0.00279676	0.50910433
chr19	56373462	NLRP4	rs12462372	G	A	0.0977011	0.00279909	0.50910433
chrX	151821277	GABRQ	rs3810651	T	A	0.511494	0.00284751	0.50910433
chr5	35037115	AGXT2	rs37369	C	T	0.155172	0.00286978	0.50910433
chr3	10302056	TATDN2	rs2241314	A	G	0.109195	0.00289537	0.50910433
chr15	59499228	LDHAL6B	rs3809529	C	T	0.333333	0.00289751	0.50910433
chr1	6693097	THAP3	rs3174820	A	G	0.344828	0.00298352	0.51564659
chr19	12384580	ZNF44	rs11879168	T	C	0.235632	0.00300828	0.51564659
chr7	6193521	USP42	rs61729726	G	C	0.252874	0.00302881	0.51564659
chr2	220046975	FAM134A	rs3731900	C	A	0.643678	0.00305453	0.51564659
chr6	90039670	UBE2J1	rs10502	G	C	0.385057	0.00313923	0.52183409
chr11	56143977	OR8U1	rs12272403	A	G	0.287356	0.00318173	0.52183409
chr9	86258685	C9orf103	rs1052690	A	C	0.155172	0.0031821	0.52183409
chr17	80789468	ZNF750	rs35653278	G	A	0.091954	0.00325388	0.52857132
chr19	45854919	ERCC2	rs13181	T	G	0.33908	0.00335982	0.54067982
chr10	73434888	CDH23	rs1227049	G	C	0.149425	0.00361299	0.56693036
chr7	50180972	C7orf72	rs1456908	C	T	0.471264	0.00363875	0.56693036
chr12	49308284	CCDC65	rs10747556	A	G	0.367816	0.00367444	0.56693036
chr12	49314994	CCDC65	rs4760600	A	G	0.367816	0.00367444	0.56693036
chr3	45869972	LZTFL1	rs1129183	C	T	0.114943	0.00372221	0.56693036
chr19	45029208	CEACAM20	rs10408247	G	A	0.091954	0.00374382	0.56693036
chr19	6156510	ACSBG2	rs33937754	A	G	0.178161	0.00386553	0.56693036
chr11	60059810	MS4A4A	rs10750931	A	G	0.178161	0.00388928	0.56693036
chr12	57390038	GPR182	rs35493121	T	C	0.0804598	0.00395611	0.56693036
chr12	57422934	MYO1A	rs17119344	G	A	0.0804598	0.00395611	0.56693036
chr4	17660082	FAM184B	rs1860596	C	T	0.672414	0.00395629	0.56693036
chr11	63072184	SLC22A10	rs72926329	C	T	0.0862069	0.00407089	0.56693036
chr4	17643848	FAM184B	rs2286771	G	A	0.666667	0.00411216	0.56693036

chr6	32609806	HLA-DQA1	rs707952	C	T	0.270115	0.00414707	0.56693036
chr10	115405615	NRAP	rs3127106	T	C	0.626437	0.00416055	0.56693036
chr10	115405664	NRAP	rs3121478	C	T	0.626437	0.00416055	0.56693036
chr8	81733727	ZNF704	rs3907424	C	A	0.143678	0.00420517	0.56693036
chr5	35039486	AGXT2	rs37370	C	T	0.83908	0.00421999	0.56693036
chr1	55248074	TTC22	rs12144325	A	G	0.126437	0.00425076	0.56693036
chr19	10961024	C19orf38	rs73009507	C	G	0.0689655	0.00427109	0.56693036
chr4	159836336	C4orf45	rs78052401	G	A	0.0517241	0.00427664	0.56693036
chr4	159780252	FNIP2	rs148251675	T	C	0.0517241	0.00427664	0.56693036
chr16	88804734	FAM38A	rs7184427	A	G	0.896552	0.00430843	0.56693036
chr18	56338792	MALT1	rs56142402	G	A	0.0862069	0.00431701	0.56693036
chr11	6524072	DNHD1	rs11605196	A	C	0.218391	0.00434606	0.56693036
chr5	176316559	HK3	rs61749653	C	T	0.0517241	0.0044251	0.56839159
chr1	169391154	C1orf114	rs3820059	A	G	0.66092	0.00444598	0.56839159
chr13	53422553	PCDH8	rs3742301	A	G	0.166667	0.00446622	0.56839159
chr6	32610009	HLA-DQA1	rs2308891	C	A	0.258621	0.0044893	0.56839159
chr16	709001	WDR90	rs4984906	C	A	0.396552	0.00457616	0.56850421
chr1	173472465	SLC9A11	rs72709309	G	A	0.0517241	0.00459557	0.56850421
chr6	1612017	FOXC1	rs398123612	A	ACGG	0.258621	0.00463856	0.56850421
chr6	1612017	FOXC1	rs755049427	A	ACGG	0.258621	0.00463856	0.56850421
chr6	1612017	HCG18	rs398123612	A	ACGG	0.258621	0.00463856	0.56850421
chr6	1612017	HCG18	rs755049427	A	ACGG	0.258621	0.00463856	0.56850421
chr19	6312290	ACER1	rs72981971	T	C	0.137931	0.00465527	0.56850421
chr1	228528563	OBSCN	rs1188710	C	G	0.534483	0.00471287	0.57108078
chr4	89319296	HERC6	rs17014118	T	C	0.16092	0.0047427	0.57108078
chr10	94695617	EXOC6	rs1326331	C	T	0.678161	0.00483042	0.57760418
chr19	45021210	CEACAM20	rs10414398	G	A	0.0804598	0.00489307	0.58096906
chr7	128119514	METTL2B	rs1065267	G	A	0.241379	0.00492604	0.58096906
chr11	7509566	OLFML1	rs12805648	A	T	0.149425	0.0050061	0.58285617
chr2	201355106	KCTD18	rs13018579	G	A	0.178161	0.00500974	0.58285617
chr14	78184566	SNW1	rs176960	C	A	0.87931	0.00521083	0.58557699

chr6	44141088	CAPN11	rs6938938	G	A	0.0689655	0.00522915	0.58557699
chr16	20802172	ERI2	rs2301770	C	T	0.0517241	0.00523108	0.58557699
chr4	84230619	HPSE	rs11099592	T	C	0.781609	0.00528825	0.58557699
chr2	220100787	ANKZF1	rs2293079	C	T	0.091954	0.00529511	0.58557699
chr21	45945648	TSPEAR	rs35028190	G	C	0.0517241	0.00537675	0.58557699
chr1	248129240	OR2AK2	rs4478844	G	A	0.603448	0.00538225	0.58557699
chr1	228494790	OBSCN	rs435776	G	A	0.488506	0.0053908	0.58557699
chr1	228504670	OBSCN	rs11810627	C	T	0.488506	0.0053908	0.58557699
chr17	47210506	B4GALNT2	rs7207403	C	A	0.603448	0.00540794	0.58557699
chr3	124627024	MUC13	rs1127233	T	G	0.241379	0.00543914	0.58557699
chr17	41361960	NBR1	rs8482	A	G	0.344828	0.00545621	0.58557699
chr10	24908686	ARHGAP21	rs3748222	T	C	0.517241	0.00549967	0.58557699
chr10	96954360	C10orf129	rs11188225	T	A	0.350575	0.00554201	0.58557699
chr3	97983391	OR5H6	rs2173236	C	G	0.155172	0.00554324	0.58557699
chr3	97983391	OR5H6	rs2173236	C	T	0.557471	0.0332514	0.72203796
chr6	167754661	TTLL2	rs909545	A	G	0.781609	0.00563395	0.58616598
chr17	10355371	MYH4	rs11651295	C	T	0.333333	0.00567678	0.58616598
chr16	705360	WDR90	rs3803697	T	C	0.402299	0.00570293	0.58616598
chr19	55174498	LILRB4	rs28366008	T	C	0.206897	0.00571795	0.58616598
chrX	151909156	CSAG1	rs2515848	A	G	0.517241	0.00580634	0.58616598
chr3	170802910	TNIK	rs17857452	C	T	0.0747126	0.00593548	0.58616598
chr5	140813	PLEKHG4B	rs73022563	G	T	0.0574713	0.00597586	0.58616598
chr16	48258198	ABCC11	rs17822931	C	T	0.172414	0.00600186	0.58616598
chr16	55897289	CES5A	rs11076126	C	T	0.0574713	0.00603215	0.58616598
chr8	2005883	MYOM2	rs17064618	C	T	0.0517241	0.00604969	0.58616598
chr7	107671407	LAMB4	rs2528693	C	G	0.0632184	0.00606606	0.58616598
chr17	64023624	CEP112	rs17704679	T	C	0.528736	0.00609735	0.58616598
chr12	49390677	DDN	rs10783299	T	C	0.367816	0.00610145	0.58616598
chr11	70118489	PPFIA1	rs546502	G	A	0.235632	0.00611955	0.58616598
chr21	47666744	MCM3AP	rs17183220	G	T	0.0747126	0.00612718	0.58616598
chr6	28264692	PGBD1	rs3800325	C	G	0.114943	0.00618624	0.58616598

chr6	28270047	PGBD1	rs6456811	C	G	0.114943	0.00618624	0.58616598
chr5	32087374	PDZD2	rs157496	A	G	0.195402	0.00619203	0.58616598
chr17	1684605	SMYD4	rs58337165	G	T	0.0517241	0.00619561	0.58616598
chrX	50350728	SHROOM4	rs143151534	T	TTCC	0.224138	0.00623563	0.58672849
chrX	50350728	SHROOM4	rs6614552	T	TTCC	0.224138	0.00623563	0.58672849
chr10	73501556	CDH23	rs1227051	G	A	0.781609	0.00636221	0.59474704
chr7	140080087	SLC37A3	rs62490396	C	G	0.425287	0.00641238	0.59474704
chr16	70896122	HYDIN	rs7192347	A	C	0.41954	0.00642447	0.59474704
chr1	175129946	KIAA0040	rs386636937	CTTCTTCTTG	T	0.275862	0.00658653	0.60648909
chr1	175129946	KIAA0040	rs2072035	CTTCTTCTTG	T	0.275862	0.00658653	0.60648909
chr1	210577901	HHAT	rs34228541	T	C	0.178161	0.00693861	0.62280979
chr7	42005678	GLI3	rs929387	G	A	0.327586	0.00716609	0.62280979
chr16	58318604	PRSS54	rs3815803	T	C	0.482759	0.00722917	0.62280979
chr19	53209553	ZNF611	rs4085566	G	A	0.436782	0.00724908	0.62280979
chr19	53209554	ZNF611	rs4085565	G	T	0.436782	0.00724908	0.62280979
chr1	93720070	CCDC18	rs12030843	C	G	0.252874	0.00728077	0.62280979
chr10	74899134	ECD	rs3736518	C	G	0.0804598	0.0073035	0.62280979
chr10	74923562	ECD	rs3812619	C	T	0.0804598	0.0073035	0.62280979
chr10	75035257	TTC18	rs4294502	T	C	0.0804598	0.0073035	0.62280979
chr5	140532	PLEKHG4B	rs12516846	T	C	0.109195	0.00730611	0.62280979
chr5	143197	PLEKHG4B	rs11949577	G	A	0.109195	0.00730611	0.62280979
chr5	143534	PLEKHG4B	rs13436090	G	A	0.109195	0.00730611	0.62280979
chr6	116600810	TSPYL1	rs3828743	G	A	0.252874	0.00735369	0.62280979
chr3	184019681	PSMD2	rs11545172	G	A	0.0747126	0.00745066	0.62280979
chr19	36017928	SBSN	rs10775583	G	C	0.385057	0.00746885	0.62280979
chr17	5436263	NLRP1	rs2301582	C	T	0.373563	0.00750947	0.62280979
chr16	88872229	CDT1	rs480727	A	G	0.431034	0.00753603	0.62280979
chr12	6639981	NCAPD2	rs2240871	C	G	0.183908	0.00757414	0.62280979
chr17	15510888	CDRT1	rs62070401	G	A	0.189655	0.00763184	0.62280979
chr17	15510988	CDRT1	rs3826385	T	C	0.189655	0.00763184	0.62280979
chr11	116691634	APOA4	rs5110	C	A	0.0689655	0.00776471	0.62280979

chr22	36122930	APOL5	rs2076671	C	T	0.333333	0.00777849	0.62280979
chr22	36124860	APOL5	rs2076673	C	G	0.333333	0.00777849	0.62280979
chr17	80895933	TBCD	rs3785522	A	G	0.775862	0.00778203	0.62280979
chr19	58879660	ZNF837	rs61746138	T	C	0.0977011	0.00779123	0.62280979
chr19	58880242	ZNF837	rs7256940	T	C	0.0977011	0.00779123	0.62280979
chr4	3006043	GRK4	rs1024323	C	T	0.316092	0.00781259	0.62280979
chr10	24873369	ARHGAP21	rs1127893	C	G	0.522989	0.00785273	0.62280979
chr15	102264476	TARSL2	rs1143136	C	A	0.235632	0.00793349	0.62280979
chr3	138191232	ESYT3	rs10935282	G	A	0.425287	0.0080507	0.62280979
chr3	53857803	CHDH	rs12676	A	C	0.672414	0.00805816	0.62280979
chr10	50122109	LRRC18	rs17772611	C	T	0.126437	0.00806064	0.62280979
chr10	50109895	WDFY4	rs35423873	A	C	0.126437	0.00806064	0.62280979
chr3	195510827	MUC4	rs413807	C	T	0.356322	0.00814807	0.62280979
chr9	98691137	C9orf102	rs2274654	T	C	0.155172	0.00816217	0.62280979
chr6	143823112	FUCA2	rs3762001	G	A	0.189655	0.00828559	0.62280979
chr9	107299001	OR13C3	rs41304943	C	A	0.114943	0.00832326	0.62280979
chr1	236175327	NID1	rs3738531	C	A	0.103448	0.00833834	0.62280979
chr22	29837566	RFPL1	rs3842466	TTCC	T	0.37931	0.00841572	0.62280979
chr22	29837566	RFPL1-AS1	rs3842466	TTCC	T	0.37931	0.00841572	0.62280979
chr19	55895588	LOC388564	rs1870074	G	A	0.833333	0.00845074	0.62280979
chr19	18255359	MAST3	rs8108738	G	A	0.517241	0.00851401	0.62280979
chr5	179290845	TBC1D9B	rs30386	T	G	0.494253	0.00852599	0.62280979
chr3	187088926	RTP4	rs1533594	G	A	0.821839	0.00854401	0.62280979
chr1	210004199	DIEXF	rs585627	C	G	0.718391	0.00858829	0.62280979
chr4	77818548	ANKRD56	rs2703129	T	C	0.373563	0.00859009	0.62280979
chr17	15496730	CDRT1	rs8078150	A	G	0.258621	0.00861401	0.62280979
chr9	115932012	FKBP15	rs57348436	G	T	0.0689655	0.00862249	0.62280979
chr9	115932150	FKBP15	rs113480096	T	TTTC	0.0689655	0.00862249	0.62280979
chr9	115933980	FKBP15	rs1128116	C	A	0.0689655	0.00862249	0.62280979
chr1	29542637	MECR	rs1128400	A	G	0.925287	0.00864461	0.62280979
chr1	29475648	SRSF4	rs2230679	T	G	0.925287	0.00864461	0.62280979

chr15	72767214	ARIH1	rs1060499844	T	TGGC	0.0574713	0.008711	0.62308375
chr21	34614250	IFNAR2	rs2229207	T	C	0.0862069	0.00874023	0.62308375
chr2	120060082	C2orf76	rs1052500	T	C	0.655172	0.00875697	0.62308375
chr19	11891003	ZNF441	rs799193	G	A	0.752874	0.00885677	0.62759145
chr4	164048199	NAF1	rs4691895	G	C	0.775862	0.00897333	0.62967947
chr4	164085425	NAF1	rs4691896	T	C	0.775862	0.00897333	0.62967947
chr18	30846895	C18orf34	rs9965081	A	T	0.931034	0.00900296	0.62967947
chr17	41223094	BRCA1	rs1799966	T	C	0.33908	0.00909226	0.62967947
chr17	41244000	BRCA1	rs16942	T	C	0.33908	0.00909226	0.62967947
chr17	41244435	BRCA1	rs16941	T	C	0.333333	0.00910565	0.62967947
chr7	97488569	ASNS	rs1049674	A	T	0.695402	0.00914362	0.62977597
chr16	31099011	PRSS53	rs11150606	T	C	0.0517241	0.00919685	0.63077675
chr9	2191309	SMARCA2	rs2296212	C	G	0.143678	0.00929727	0.63077675
chr9	18777196	ADAMTSL1	rs41268983	A	C	0.114943	0.00932881	0.63077675
chr5	80409526	RASGRF2	rs34193571	T	C	0.0862069	0.00932948	0.63077675
chr17	41244936	BRCA1	rs799917	G	A	0.350575	0.00950292	0.63077675
chr18	54815025	BOD1P	rs11151997	G	T	0.574713	0.0095504	0.63077675
chr18	44181227	LOXHD1	rs10163657	C	T	0.091954	0.0095818	0.63077675
chr19	17451981	GTPBP3	rs3745193	G	A	0.0574713	0.00959429	0.63077675
chr19	35422808	ZNF30	rs10422961	C	T	0.247126	0.00963903	0.63077675
chr17	62020348	SCN4A	rs2058194	T	C	0.5	0.00969879	0.63077675
chr1	248128929	OR2AK2	rs6664332	G	A	0.367816	0.00972643	0.63077675
chr1	9324213	H6PD	rs17368528	C	T	0.103448	0.00981585	0.63077675
chr1	151735576	MRPL9	rs7007	T	C	0.137931	0.00984056	0.63077675
chr1	151735576	OAZ3	rs7007	T	C	0.137931	0.00984056	0.63077675
chr2	133174764	GPR39	rs2241764	C	T	0.367816	0.00989613	0.63077675
chr5	149546828	CDX1	rs2302275	C	G	0.471264	0.00995655	0.63077675
chr16	89291210	ZNF778	rs10625512	G	GGTGA	0.885057	0.00996191	0.63077675
chr11	6541225	DNHD1	rs11603869	C	G	0.235632	0.0099647	0.63077675
chr3	58625875	FAM3D	rs33966924	C	A	0.149425	0.0099893	0.63077675
chr4	101108875	DDIT4L	rs58706659	GT	G	0.114943	0.00999469	0.63077675

chr4	101108877	DDIT4L	rs201713115	T	C	0.114943	0.00999469	0.63077675
chr6	2685775	HCG27	rs34953021	G	A	0.091954	0.0100007	0.63077675
chr6	2685775	MYLK4	rs34953021	G	A	0.091954	0.0100007	0.63077675
chr15	85188839	WDR73	rs11073619	C	T	0.103448	0.0100862	0.63203122
chr17	17896205	LRRC48	rs4584886	C	T	0.293103	0.010094	0.63203122
chr11	8132301	RIC3	rs11826236	C	T	0.0804598	0.0102241	0.63347405
chr11	8159857	RIC3	rs55990541	C	T	0.0804598	0.0102241	0.63347405
chr12	106641489	CKAP4	rs368519473	GGGC	G	0.614943	0.0102352	0.63347405
chr11	117267884	CEP164	rs573455	A	G	0.511494	0.0102642	0.63347405
chr11	102477377	MMP20	rs1784424	G	T	0.511494	0.0104716	0.64167431
chr11	102477395	MMP20	rs1784423	A	G	0.511494	0.0104716	0.64167431
chr19	51485622	KLK7	rs2659067	A	G	0.862069	0.0105438	0.64183062
chr1	203186093	CHIT1	rs1065761	G	C	0.12069	0.0105487	0.64183062
chr4	36310065	DTHD1	rs61739432	G	A	0.0574713	0.0106311	0.64456659
chr2	148730367	ORC4	rs2307397	G	C	0.0574713	0.0109095	0.64661247
chr19	35435006	ZNF30	rs1345658	G	A	0.436782	0.0110277	0.64661247
chr19	35450229	ZNF792	rs2651079	C	T	0.436782	0.0110277	0.64661247
chr16	48250026	ABCC11	rs11863236	G	T	0.0574713	0.0110745	0.64661247
chr16	48265777	ABCC11	rs16945988	C	T	0.0574713	0.0110745	0.64661247
chr19	35434238	ZNF30	rs1811	A	G	0.431034	0.0110947	0.64661247
chr20	37396120	ACTR5	rs2245231	A	G	0.413793	0.0111281	0.64661247
chr22	29837537	RFPL1	rs3804076	T	C	0.367816	0.0112667	0.64661247
chr22	29837537	RFPL1-AS1	rs3804076	T	C	0.367816	0.0112667	0.64661247
chr7	6194274	USP42	rs6463529	T	C	0.126437	0.0114191	0.64661247
chr2	29274730	FAM179A	rs7577483	G	T	0.091954	0.0114219	0.64661247
chr8	72936145	TRPA1	rs959976	T	C	0.183908	0.0115374	0.64661247
chr19	51843808	VSIG10L	rs7259266	C	T	0.614943	0.0116371	0.64661247
chr19	51845371	VSIG10L	rs10414211	T	G	0.614943	0.0116371	0.64661247
chr7	2649777	IQCE	rs1061566	C	T	0.137931	0.0117305	0.64661247
chr6	29796376	HLA-G	rs12722477	C	A	0.0977011	0.0117582	0.64661247
chr6	44115169	TMEM63B	rs4714759	G	A	0.0977011	0.0118422	0.64661247

chr4	15709192	BST1	rs2302465	G	A	0.149425	0.0119245	0.64661247
chr4	15688604	FAM200B	rs2302469	G	C	0.149425	0.0119245	0.64661247
chr19	49376584	PPP1R15A	rs3786734	G	A	0.0747126	0.0119568	0.64661247
chr16	722331	RHOT2	rs3177338	C	T	0.408046	0.0119643	0.64661247
chr16	715990	WDR90	rs7190775	G	A	0.396552	0.0120736	0.64661247
chr5	139931628	SRA1	.	A	AGTC	0.448276	0.0120779	0.64661247
chr5	139931629	SRA1	rs202193903	C	G	0.448276	0.0120779	0.64661247
chr6	29408313	OR10C1	rs2074466	C	A	0.178161	0.0120799	0.64661247
chr6	29394926	OR11A1	rs9257857	C	T	0.178161	0.0120799	0.64661247
chr8	20038466	SLC18A1	rs2270641	T	G	0.413793	0.0120917	0.64661247
chr11	4967532	OR51A4	rs2595988	G	C	0.16092	0.0120941	0.64661247
chr3	130947435	NEK11	rs3738000	A	T	0.718391	0.0121129	0.64661247
chr1	152128212	RPTN	rs143744326	C	G	0.0574713	0.0121152	0.64661247
chr1	29475341	SRSF4	rs2230678	C	T	0.833333	0.0121369	0.64661247
chr1	29475394	SRSF4	rs2230677	C	G	0.833333	0.0121369	0.64661247
chr1	43201614	CLDN19	rs9660973	C	T	0.0517241	0.012144	0.64661247
chr1	43212926	LEPRE1	rs67014447	G	A	0.0517241	0.012144	0.64661247
chr1	43223489	LEPRE1	rs6700677	C	T	0.0517241	0.012144	0.64661247
chr1	43232504	LEPRE1	rs55716016	C	A	0.0517241	0.012144	0.64661247
chr17	10541515	MYH3	rs2285477	C	T	0.810345	0.0121512	0.64661247
chr11	26586801	MUC15	rs15783	G	A	0.655172	0.0122031	0.64661247
chr19	54745907	LILRA6	rs62133127	A	G	0.189655	0.0122369	0.64661247
chr2	29256374	FAM179A	rs60403047	C	T	0.091954	0.0122507	0.64661247
chr6	157100396	ARID1B	rs572236007	G	GCGC	0.091954	0.0122815	0.64661247
chr5	174106	PLEKHG4B	rs4956987	G	A	0.574713	0.0123224	0.64661247
chr1	55247097	TTC22	rs2286203	G	A	0.172414	0.0123643	0.64661247
chr11	5718517	TRIM22	rs7935564	G	A	0.609195	0.0123777	0.64661247
chr21	35239562	ITSN1	rs56279221	A	G	0.103448	0.012421	0.64661247
chr15	45554267	SLC28A2	rs1060896	C	A	0.637931	0.0124235	0.64661247
chr14	105416220	AHNAK2	rs2819435	T	A	0.764368	0.0124298	0.64661247
chr4	169928842	CBR4	rs2877380	G	T	0.729885	0.0125286	0.64978905

chr2	174128513	ZAK	rs3769148	C	T	0.488506	0.0127651	0.65957534
chr17	46939658	CALCOCO2	rs10278	C	G	0.316092	0.0127939	0.65957534
chr6	34831856	UHRF1BP1	rs13205210	T	C	0.0862069	0.0128635	0.6611839
chr19	9024871	MUC16	rs62118272	C	T	0.0862069	0.0129539	0.66301836
chr7	107677984	LAMB4	rs1627354	G	A	0.0632184	0.0129762	0.66301836
chr17	78091405	GAA	rs1126690	G	A	0.758621	0.0130254	0.6635632
chr6	133045812	VNN3	rs2294758	G	A	0.316092	0.0130901	0.66489213
chr11	62434173	METTL12	rs11231181	G	A	0.275862	0.0131538	0.66616259
chr1	197070521	ASPM	rs12138336	C	G	0.0574713	0.0131956	0.66631975
chr6	130374102	L3MBTL3	rs9388768	C	A	0.678161	0.0132944	0.66934583
chr8	2021421	MYOM2	rs2272720	G	T	0.431034	0.0133468	0.66978907
chr2	113498566	CKAP2L	rs3811040	A	G	0.545977	0.013381	0.66978907
chr15	59981590	BNIP2	rs754641	G	A	0.45977	0.0134852	0.67113906
chr20	44005936	TP53TG5	rs2231616	C	T	0.132184	0.0135224	0.67113906
chr2	84848596	DNAH6	rs61733547	G	A	0.0804598	0.0135249	0.67113906
chr19	12186732	ZNF844	rs76842919	A	G	0.0747126	0.0135886	0.67236237
chr1	64095111	PGM1	rs855314	A	G	0.137931	0.0136444	0.67318889
chr19	9868278	ZNF846	rs10420364	T	C	0.206897	0.0137312	0.6736112
chr19	9868404	ZNF846	rs10414485	C	T	0.206897	0.0137312	0.6736112
chr16	2260612	C16orf79	rs26856	T	C	0.689655	0.0140514	0.67921001
chr20	18794754	C20orf79	rs1053839	C	T	0.16092	0.0140638	0.67921001
chr6	30313340	RPP21	rs6986	G	C	0.258621	0.0141323	0.67921001
chr6	30313340	TRIM39-RPP21	rs6986	G	C	0.258621	0.0141323	0.67921001
chr11	62439569	C11orf83	rs13941	G	A	0.695402	0.0141434	0.67921001
chr10	101147692	CNNM1	rs2298316	G	A	0.091954	0.0141744	0.67921001
chr2	120078778	C2orf76	rs1132267	T	C	0.666667	0.0142211	0.67921001
chr19	9028373	MUC16	rs56396897	G	A	0.281609	0.0142923	0.67921001
chr7	99457605	CYP3A43	rs680055	C	G	0.091954	0.0143338	0.67921001
chr7	99473858	OR2AE1	rs17161997	G	C	0.091954	0.0143338	0.67921001
chr10	70856852	SRGN	rs2229498	G	A	0.804598	0.0144273	0.67921001

chr8	92136728	LRRC69	rs11785003	C	T	0.563218	0.0144284	0.67921001
chr8	72977703	TRPA1	rs920829	C	T	0.126437	0.0144383	0.67921001
chr2	74007136	DUSP11	rs2272051	T	C	0.649425	0.0144799	0.67921001
chr1	20246876	PLA2G2E	rs61729970	C	T	0.109195	0.0144838	0.67921001
chr19	56953963	ZNF667	rs35914474	G	A	0.494253	0.0144923	0.67921001
chr6	143823157	FUCA2	rs3762002	T	C	0.195402	0.0145159	0.67921001
chr6	31379931	MICA	rs1063635	G	A	0.5	0.0146552	0.68339063
chr16	3165425	MGC3771	rs909410	A	G	0.413793	0.0147439	0.68339063
chr16	3165425	ZNF205	rs909410	A	G	0.413793	0.0147439	0.68339063
chr19	3821408	ZFR2	rs61742027	G	A	0.0517241	0.014764	0.68339063
chr2	71160222	VAX2	rs2234500	C	G	0.091954	0.0150018	0.69097913
chr15	90335788	ANPEP	rs25651	C	T	0.333333	0.0150082	0.69097913
chr10	26434455	MYO3A	rs33947968	G	T	0.12069	0.0150857	0.69269512
chr19	44352665	ZNF283	rs2356437	T	C	0.735632	0.0152004	0.69498064
chr12	48151822	RAPGEF3	rs11168230	C	G	0.321839	0.0152162	0.69498064
chr9	100388119	TSTD2	rs10817858	G	T	0.218391	0.0153323	0.69498064
chr14	105411700	AHNAK2	rs4264326	A	G	0.494253	0.0153493	0.69498064
chr12	12483764	MANSC1	rs17375215	C	T	0.0574713	0.0153539	0.69498064
chr19	56953585	ZNF667	rs3760849	T	C	0.494253	0.0153852	0.69498064
chr21	45811343	TRPM2	rs1556314	T	G	0.206897	0.0154784	0.69498064
chr1	144879339	PDE4DIP	rs145959230	C	T	0.0804598	0.015498	0.69498064
chr17	3627473	GSG2	rs9907144	C	T	0.477011	0.0155621	0.69498064
chr17	3628362	GSG2	rs3809806	T	C	0.477011	0.0155621	0.69498064
chr18	11689699	GNAL	rs7236433	G	A	0.172414	0.0155929	0.69498064
chr1	62673225	L1TD1	rs7533274	G	A	0.649425	0.0156402	0.69498064
chr2	75923413	C2orf3	rs7560262	T	C	0.45977	0.0156796	0.69498064
chr17	2600186	KIAA0664	rs11078312	G	A	0.137931	0.0157863	0.69498064
chr6	116575116	TSPYL4	rs17524614	G	T	0.137931	0.0157974	0.69498064
chr1	179852074	TOR1AIP1	rs1281378	T	C	0.609195	0.0158205	0.69498064
chr1	179876988	TOR1AIP1	rs609521	C	G	0.603448	0.0158885	0.69498064
chr8	63951312	GGH	rs1800909	A	G	0.275862	0.015906	0.69498064

chr16	71660404	MARVELD3	rs61753635	G	A	0.298851	0.0159221	0.69498064
chr19	17392630	ANKLE1	rs139615873	G	GC	0.0689655	0.0159427	0.69498064
chr19	45867259	ERCC2	rs1799793	C	T	0.321839	0.0160467	0.6977478
chr14	105411781	AHNAK2	rs10438247	G	A	0.494253	0.0161895	0.69823686
chr19	8486884	MARCH2	rs1133893	G	A	0.275862	0.0162561	0.69823686
chr17	67210992	ABCA10	rs11657804	T	C	0.402299	0.0162823	0.69823686
chr19	464310	ODF3L2	rs34551779	A	G	0.482759	0.0162912	0.69823686
chr6	31378358	MICA	rs1063630	T	G	0.189655	0.0163048	0.69823686
chr6	31380198	MICA	rs61738275	C	T	0.189655	0.0163048	0.69823686
chr16	58743454	GOT2	rs30842	A	C	0.66092	0.0163418	0.69823686
chr19	22156943	ZNF208	rs12462668	G	A	0.126437	0.0164305	0.69829798
chr1	202129826	PTPN7	rs4309039	T	C	0.431034	0.0164859	0.69829798
chr22	18923713	PRODH	rs3815655	G	A	0.0747126	0.0165272	0.69829798
chr1	215914826	USH2A	rs35309576	T	C	0.264368	0.016546	0.69829798
chr1	215916563	USH2A	rs11120616	G	A	0.264368	0.016546	0.69829798
chr15	43571463	TGM7	rs148818002	G	A	0.0574713	0.0166278	0.69878482
chr8	63951237	GGH	rs11545077	C	T	0.241379	0.0166387	0.69878482
chr14	105408811	AHNAK2	rs2819421	A	G	0.494253	0.0167011	0.69969888
chr12	1910786	CACNA2D4	rs62621429	C	T	0.0632184	0.0168126	0.70123136
chr19	33647379	WDR88	rs11881580	T	C	0.12069	0.0168437	0.70123136
chr6	31380194	MICA	rs41554412	T	C	0.16092	0.0168905	0.70123136
chr4	84383810	FAM175A	rs12642536	C	T	0.408046	0.0169305	0.70123136
chr16	708275	WDR90	rs45613635	C	A	0.224138	0.0169413	0.70123136
chr14	105418391	AHNAK2	rs11625007	C	T	0.488506	0.0170417	0.70299648
chr19	7763248	FCER2	rs2228137	G	A	0.143678	0.0171074	0.70299648
chr5	127609633	FBN2	rs2291628	G	A	0.0747126	0.0171948	0.70299648
chr17	16664991	CCDC144A	rs3869489	G	A	0.0747126	0.0173281	0.70299648
chr16	88808743	FAM38A	rs6500495	A	G	0.925287	0.0173345	0.70299648
chr5	179264731	C5orf45	rs10277	T	C	0.563218	0.0174135	0.70299648
chr5	179264731	SQSTM1	rs10277	T	C	0.563218	0.0174135	0.70299648
chr5	179280379	C5orf45	rs1650893	T	C	0.563218	0.0174135	0.70299648

chr11	60070176	MS4A4A	rs6591561	A	G	0.310345	0.0175306	0.70299648
chr4	967191	DGKQ	rs17855876	G	A	0.091954	0.0176471	0.70299648
chr1	34038214	CSMD2	rs2641962	T	C	0.436782	0.0178639	0.70299648
chr7	140244560	DENND2A	rs2293177	C	T	0.333333	0.0179341	0.70299648
chr20	62193445	PRIC285	rs3810481	G	A	0.172414	0.0180148	0.70299648
chr3	121415610	GOLGB1	rs33988592	G	A	0.287356	0.0180304	0.70299648
chr3	121351338	HCLS1	rs2070180	C	T	0.287356	0.0180304	0.70299648
chr10	115409840	NRAP	rs2275799	C	T	0.298851	0.018043	0.70299648
chr9	117808785	TNC	rs2104772	T	A	0.471264	0.0182558	0.70299648
chr7	73097720	DNAJC30	rs28494095	G	C	0.126437	0.0183843	0.70299648
chr1	153748161	SLC27A3	rs34527123	G	C	0.0747126	0.0185107	0.70299648
chr7	92760738	SAMD9L	rs10282508	T	G	0.0574713	0.0185247	0.70299648
chr1	158725080	OR6K6	rs16841001	C	T	0.12069	0.0185842	0.70299648
chr3	40503506	RPL14	rs148931839	C	T	0.0632184	0.0186022	0.70299648
chr11	64981587	SLC22A20	rs11605632	G	A	0.408046	0.018664	0.70299648
chr20	23860178	CST5	rs1799841	A	G	0.390805	0.0186865	0.70299648
chr1	111857208	CHIA	rs3818822	G	A	0.103448	0.0186935	0.70299648
chr17	10346781	MYH4	rs3744554	T	C	0.396552	0.0187121	0.70299648
chr17	10355763	MYH4	rs917361	T	C	0.396552	0.0187121	0.70299648
chr17	10297658	MYH8	rs8069834	A	G	0.603448	0.0187161	0.70299648
chr6	34214322	C6orf1	rs1150781	C	G	0.87931	0.0187236	0.70299648
chr17	46607958	HOXB1	rs12939811	T	A	0.149425	0.0187718	0.70299648
chr4	73414286	ADAMTS3	rs788908	C	T	0.649425	0.0189156	0.70299648
chr17	78079597	GAA	rs1042393	A	G	0.752874	0.0189339	0.70299648
chr17	78079669	GAA	rs1042395	G	A	0.752874	0.0189339	0.70299648
chr10	46965018	SYT15	rs3127785	C	G	0.454023	0.0190104	0.70299648
chr16	863355	PRR25	rs138733834	AGGACGCCGG ACCGACAC	A	0.0804598	0.0190959	0.70299648
chr8	27925204	C8orf80	rs4732620	T	C	0.568966	0.0191167	0.70299648
chr20	139456	DEFB127	rs12624954	G	A	0.385057	0.0191313	0.70299648
chr20	139576	DEFB127	rs16995685	C	A	0.385057	0.0191313	0.70299648

chr1	115231254	AMPD1	rs61752479	G	A	0.0977011	0.0191471	0.70299648
chr21	47766113	PCNT	rs34500739	T	G	0.0689655	0.0191904	0.70299648
chr21	47831845	PCNT	rs34268261	G	A	0.0689655	0.0191904	0.70299648
chr21	47841989	PCNT	rs61735814	C	T	0.0689655	0.0191904	0.70299648
chr21	47851636	PCNT	rs743346	G	A	0.0689655	0.0191904	0.70299648
chr21	47717493	YBEY	rs61736728	G	T	0.0689655	0.0191904	0.70299648
chr20	31627291	BPIL3	rs4911287	A	G	0.442529	0.019219	0.70299648
chr21	40720494	HMGN1	rs34613278	C	T	0.0632184	0.0192225	0.70299648
chr11	47788663	FNBP4	rs397711020	CGGTGGT	C	0.45977	0.0193119	0.70299648
chr7	149499284	SSPO	rs56163742	C	T	0.126437	0.0193288	0.70299648
chr5	33998768	C1QTNF3-AMACR	rs34677	C	A	0.143678	0.0193997	0.70299648
chr5	33998768	AMACR	rs34677	C	A	0.143678	0.0193997	0.70299648
chr9	15188106	TTC39B	rs1407977	T	C	0.649425	0.019429	0.70299648
chr11	47701528	AGBL2	rs12286721	C	A	0.517241	0.019452	0.70299648
chr10	100017453	LOXL4	rs1983864	T	G	0.321839	0.0195089	0.70299648
chr6	26505362	BTN1A1	rs3736781	G	A	0.528736	0.0195136	0.70299648
chr6	26509330	BTN1A1	rs9393728	C	G	0.528736	0.0195136	0.70299648
chr14	105416959	AHNAK2	rs2013462	A	G	0.534483	0.019627	0.70299648
chr19	18273047	PIK3R2	rs1011320	T	C	0.925287	0.0197351	0.70299648
chr3	123665902	CCDC14	rs17310144	T	G	0.597701	0.0197368	0.70299648
chr10	115348046	HABP2	rs7080536	G	A	0.0632184	0.0197806	0.70299648
chr18	43246156	SLC14A2	rs9960464	G	A	0.448276	0.0197938	0.70299648
chr14	23830042	EFS	rs2231798	T	C	0.281609	0.0197947	0.70299648
chr1	17708528	PADI6	rs61766772	G	A	0.0747126	0.0198006	0.70299648
chr2	29225504	FAM179A	rs72786179	T	C	0.0632184	0.0198129	0.70299648
chr1	231408091	GNPAT	rs11558492	A	G	0.172414	0.0198659	0.70299648
chr14	105418344	AHNAK2	rs55791176	T	G	0.477011	0.0198668	0.70299648
chr9	97055310	ZNF169	rs1536690	C	T	0.183908	0.0199175	0.70299648
chr6	28269663	PGBD1	rs1997660	A	G	0.316092	0.0199812	0.70299648
chr15	59981515	BNIP2	rs3087328	A	G	0.465517	0.0199894	0.70299648

chr19	17000632	F2RL3	rs773902	G	A	0.224138	0.020015	0.70299648
chr22	38477275	SLC16A8	rs75640043	G	A	0.0689655	0.0200365	0.70299648
chr2	174131392	ZAK	rs35608243	T	C	0.091954	0.0200567	0.70299648
chr2	88472791	THNSL2	rs4129190	G	A	0.827586	0.0200629	0.70299648
chr7	1028448	CYP2W1	rs3808348	C	T	0.178161	0.0202043	0.70299648
chr12	9317784	PZP	rs2277413	A	G	0.229885	0.0202629	0.70299648
chr11	299411	IFITM5	rs57285449	C	G	0.413793	0.0203131	0.70299648
chr16	88807896	FAM38A	rs7404939	G	A	0.0574713	0.020413	0.70299648
chr1	20490518	PLA2G2C	rs6426616	T	C	0.574713	0.0204456	0.70299648
chr1	2441358	PANK4	rs2494620	T	C	0.316092	0.0204583	0.70299648
chr19	41497274	CYP2B6	rs8192709	C	T	0.0574713	0.0205385	0.70299648
chr14	65253232	SPTB	rs77806	T	C	0.270115	0.0206414	0.70299648
chr10	105177645	PDCD11	rs11598673	G	T	0.0574713	0.0207201	0.70299648
chr18	44561100	TCEB3B	rs2571028	C	G	0.557471	0.0207833	0.70299648
chr14	94936110	SERPINA9	rs45438398	G	A	0.12069	0.0208754	0.70299648
chr19	9225685	OR7G1	rs2195951	T	C	0.183908	0.0209601	0.70299648
chr19	9226192	OR7G1	rs6511874	A	G	0.183908	0.0209601	0.70299648
chr22	42336172	CENPM	rs5758511	G	A	0.270115	0.0209813	0.70299648
chr14	105414790	AHNAK2	rs2582513	A	G	0.505747	0.0210001	0.70299648
chr3	124728626	HEG1	rs6438869	A	G	0.655172	0.021001	0.70299648
chr16	68719113	CDH3	rs34494880	G	A	0.0689655	0.0210058	0.70299648
chr5	5146335	ADAMTS16	rs2086310	C	G	0.83908	0.0211061	0.70299648
chr14	105405599	AHNAK2	rs3742935	G	C	0.5	0.0211156	0.70299648
chr14	105406238	AHNAK2	rs2819419	A	C	0.5	0.0211156	0.70299648
chr14	105407798	AHNAK2	rs4465542	T	C	0.5	0.0211156	0.70299648
chr14	105408955	AHNAK2	rs2819422	A	G	0.5	0.0211156	0.70299648
chr14	105410183	AHNAK2	rs10438246	T	C	0.5	0.0211156	0.70299648
chr14	105410827	AHNAK2	rs28380382	C	T	0.5	0.0211156	0.70299648
chr14	105414629	AHNAK2	rs72702027	G	A	0.5	0.0211156	0.70299648
chr14	105393556	PLD4	rs2841280	G	C	0.5	0.02112	0.70299648
chr17	18024266	MYO15A	rs2955367	T	G	0.275862	0.0211649	0.70299648

chr10	12143105	DHTKD1	rs2062988	C	G	0.810345	0.0212307	0.70299648
chr1	117122288	IGSF3	rs647711	G	C	0.442529	0.0213975	0.70299648
chr1	117122288	IGSF3	rs781349066	G	C	0.442529	0.0213975	0.70299648
chr1	156264648	C1orf85	rs1570805	C	T	0.091954	0.0214234	0.70299648
chr2	241451351	ANKMY1	rs35044862	G	A	0.12069	0.0214537	0.70299648
chr13	39343822	FREM2	rs9603422	C	T	0.103448	0.0215874	0.70299648
chr3	98002587	OR5H2	rs16839611	A	G	0.247126	0.0215891	0.70299648
chr16	31374535	ITGAX	rs2230429	C	G	0.321839	0.0216833	0.70299648
chr17	79089590	BAIAP2	rs4969391	A	G	0.201149	0.0217062	0.70299648
chr3	111887792	SLC9A10	rs76007436	C	A	0.0977011	0.0217063	0.70299648
chr6	2955802	SERPINB6	rs2295769	T	C	0.310345	0.0217462	0.70299648
chr6	2955802	BAG6	rs2295769	T	C	0.310345	0.0217462	0.70299648
chr6	2955802	APOM	rs2295769	T	C	0.310345	0.0217462	0.70299648
chr10	18828635	CACNB2	rs58225473	T	G	0.155172	0.0218274	0.70299648
chr7	30831082	FAM188B	rs12701034	C	T	0.431034	0.0218528	0.70299648
chr7	30831082	INMT-FAM188B	rs12701034	C	T	0.431034	0.0218528	0.70299648
chrX	111698440	ZCCHC16	rs7474140	G	T	0.183908	0.0218675	0.70299648
chr3	142178144	ATR	rs2229032	C	T	0.149425	0.021917	0.70299648
chr14	105408827	AHNAK2	rs11850949	A	C	0.5	0.0219672	0.70299648
chr11	309127	IFITM2	rs1059091	A	G	0.356322	0.0219754	0.70299648
chr22	43572354	TTLL12	rs11704935	C	T	0.12069	0.0219817	0.70299648
chr6	90448092	MDN1	rs4140446	C	T	0.856322	0.0220058	0.70299648
chr19	12921186	RNASEH2A	rs7247284	T	C	0.0517241	0.0220499	0.70299648
chr15	85401259	ALPK3	rs306197	C	T	0.752874	0.022079	0.70299648
chr2	241569692	GPR35	rs3749171	C	T	0.16092	0.0220827	0.70299648
chr5	140559320	PCDHB8	rs17844498	G	A	0.408046	0.0220908	0.70299648
chr12	971291	WNK1	rs2286007	C	T	0.0977011	0.0221186	0.70299648
chr10	102770082	PDZD7	rs807023	T	G	0.810345	0.0221517	0.70299648
chr19	5893058	NDUFA11	rs12980262	G	A	0.126437	0.0222186	0.70299648
chr11	47640429	MTCH2	rs1064608	G	C	0.304598	0.0222371	0.70299648

chr5	44809162	MRPS30	rs3747479	G	C	0.373563	0.0222506	0.70299648
chr6	83838673	DOPEY1	rs4706980	G	A	0.132184	0.0223728	0.70531915
chr3	137787047	DZIP1L	rs374045	C	T	0.87931	0.022447	0.70531915
chr3	137787192	DZIP1L	rs446644	T	C	0.87931	0.022447	0.70531915
chr10	99531571	SFRP5	rs11815012	C	G	0.804598	0.0225328	0.70672547
chr10	51568378	NCOA4	rs10761581	T	G	0.373563	0.0226283	0.70843036
chr10	115393929	NRAP	rs3189030	G	A	0.304598	0.0226868	0.70853883
chr2	167279922	SCN7A	rs6738031	C	A	0.672414	0.0227405	0.70853883
chrX	23928489	CXorf58	rs2707164	C	T	0.528736	0.0228153	0.70853883
chr11	56409603	OR5AP2	rs11606499	C	T	0.103448	0.0228742	0.70853883
chr17	80789628	ZNF750	rs8074277	T	C	0.218391	0.0229271	0.70853883
chr12	53227803	KRT79	rs2638497	A	G	0.545977	0.0229663	0.70853883
chr3	98110406	OR5K3	rs144759043	G	GA	0.557471	0.0230105	0.70853883
chr11	118890022	TRAPPC4	rs3802881	C	T	0.0977011	0.0230357	0.70853883
chr2	152322095	RIF1	rs2444257	A	T	0.678161	0.0230456	0.70853883
chr2	152331418	RIF1	rs1065177	C	G	0.678161	0.0230456	0.70853883
chr17	17696755	RAI1	rs11649804	C	A	0.327586	0.0230844	0.70853883
chr12	104139034	STAB2	rs7306642	C	A	0.0977011	0.0232952	0.71373674
chr3	37476427	C3orf35	rs17266511	C	T	0.0862069	0.0233737	0.71417739
chr2	167334085	SCN7A	rs7565062	G	T	0.683908	0.0235027	0.71417739
chr1	159854251	CCDC19	rs16842789	T	C	0.091954	0.0235072	0.71417739
chr9	100388197	TSTD2	rs2773347	C	T	0.637931	0.0235395	0.71417739
chr17	56584508	MTMR4	rs2302190	T	C	0.206897	0.0237162	0.71417739
chr17	56618030	SEPT4	rs8073754	C	T	0.206897	0.0237162	0.71417739
chr13	52603896	UTP14C	rs17402034	G	A	0.109195	0.0237318	0.71417739
chr11	62294309	AHNAK	rs141117375	C	T	0.0574713	0.0237928	0.71417739
chr2	29416481	ALK	rs1881420	T	C	0.252874	0.0238516	0.71417739
chr1	159752066	DUSP23	rs1129923	G	A	0.109195	0.0238635	0.71417739
chr10	72500763	ADAMTS14	rs10823607	T	C	0.804598	0.0239006	0.71417739
chr17	17696531	RAI1	rs3803763	G	C	0.333333	0.0239154	0.71417739
chr8	120429024	NOV	rs2279112	G	A	0.264368	0.0239246	0.71417739

chr10	81371729	SFTPA1	rs1136450	C	G	0.591954	0.0239518	0.71417739
chr10	115392919	NRAP	rs2270182	T	A	0.293103	0.0239705	0.71417739
chr17	27044482	RAB34	rs11545700	C	A	0.149425	0.0239732	0.71417739
chr11	4967468	OR51A4	rs28698374	G	A	0.821839	0.0240361	0.71481452
chr16	863498	PRR25	rs13333991	G	C	0.0517241	0.0241739	0.71500718
chr1	12854530	PRAMEF1	rs1063776	C	G	0.87931	0.0241786	0.71500718
chr10	101977883	CHUK	rs2230804	C	T	0.454023	0.0242217	0.71500718
chr7	126542667	GRM8	rs78124913	A	T	0.132184	0.0242387	0.71500718
chr6	152469188	SYNE1	rs2252755	C	G	0.37931	0.0242502	0.71500718
chr2	43452618	ZFP36L2	rs11675632	C	T	0.0517241	0.0243463	0.71506707
chr2	159663616	DAPL1	rs12535	G	A	0.321839	0.024569	0.71506707
chr22	21384578	SLC7A4	rs2270384	C	T	0.091954	0.0246491	0.71506707
chr22	21386019	SLC7A4	rs2072550	G	A	0.091954	0.0246491	0.71506707
chr18	44560429	TCEB3B	rs892586	C	A	0.563218	0.0246616	0.71506707
chr17	39619186	KRT32	rs2604956	G	C	0.114943	0.0246889	0.71506707
chr11	123624658	OR6X1	rs12364099	G	T	0.166667	0.0247018	0.71506707
chr11	66468469	SPTBN2	rs506028	A	G	0.0689655	0.0247503	0.71506707
chr22	31535995	PLA2G3	rs2074734	C	G	0.0689655	0.024874	0.71506707
chr16	2155426	PKD1	rs9936785	T	C	0.126437	0.0249669	0.71506707
chr2	101925026	RNF149	rs11123868	T	C	0.37931	0.0250253	0.71506707
chr17	39659913	KRT13	rs9891361	G	A	0.885057	0.0250258	0.71506707
chr12	52631313	KRT7	rs6580870	A	G	0.833333	0.0250769	0.71506707
chr16	14988868	NOMO1	rs62038492	A	G	0.454023	0.0251857	0.71506707
chr19	3820191	ZFR2	rs2301843	C	T	0.362069	0.0252538	0.71506707
chr19	10224526	P2RY11	rs3745600	C	T	0.436782	0.0252685	0.71506707
chr19	10224526	PPAN-P2RY11	rs3745600	C	T	0.436782	0.0252685	0.71506707
chr1	117656088	TRIM45	rs1289658	A	G	0.408046	0.0254331	0.71506707
chr14	21215923	EDDM3A	rs34552133	G	T	0.0977011	0.0254787	0.71506707
chr17	46608184	HOXB1	rs145570960	G	GGGGCGCTGT	0.149425	0.0255473	0.71506707
chr19	35757427	LSR	rs34259399	G	A	0.126437	0.0256257	0.71506707
chr5	36985303	NIPBL	rs3822471	A	G	0.091954	0.0257093	0.71506707

chr7	149462381	ZNF467	rs112589121	C	T	0.0689655	0.0257547	0.71506707
chr5	146460691	PPP2R2B	rs17524553	T	A	0.114943	0.0258239	0.71506707
chr8	27308585	PTK2B	rs751019	A	C	0.408046	0.0258522	0.71506707
chr19	50360989	PTOV1	rs59595912	A	G	0.0689655	0.0258656	0.71506707
chr10	71164655	TACR2	rs2229170	C	T	0.178161	0.0260823	0.71506707
chr17	4461748	GGT6	rs7215121	G	A	0.396552	0.0260884	0.71506707
chr16	53481010	RBL2	rs17800727	A	G	0.264368	0.0261584	0.71506707
chr6	28294550	ZNF323	rs853684	T	C	0.367816	0.0262389	0.71506707
chr19	43244485	PSG3	rs11880551	G	C	0.132184	0.0262941	0.71506707
chr1	154516477	TDRD10	rs12750774	G	A	0.281609	0.0263	0.71506707
chr1	202183308	LGR6	rs10920362	C	T	0.477011	0.0263405	0.71506707
chr20	3204084	ITPA	rs9101	G	A	0.344828	0.0263945	0.71506707
chr1	21024901	KIF17	rs522496	C	T	0.678161	0.0264099	0.71506707
chr3	128204951	GATA2	rs2335052	C	T	0.172414	0.0264425	0.71506707
chr3	122296647	PARP15	rs7632426	G	A	0.091954	0.0264664	0.71506707
chr7	16460893	ISPD	rs7782939	T	G	0.304598	0.02647	0.71506707
chr17	18023897	MYO15A	rs2955365	G	A	0.264368	0.0264702	0.71506707
chr11	60718792	SLC15A3	rs150370599	C	T	0.0862069	0.0265062	0.71506707
chr9	113196733	SVEP1	rs7852962	A	C	0.155172	0.0265499	0.71506707
chr9	113221247	SVEP1	rs7038903	T	C	0.155172	0.0265499	0.71506707
chr1	165532746	LRRC52	rs17407838	T	A	0.114943	0.02657	0.71506707
chr19	6901891	EMR1	rs897738	G	A	0.252874	0.0266954	0.71506707
chr19	6903920	EMR1	rs443658	A	G	0.252874	0.0266954	0.71506707
chr19	6904137	EMR1	rs370094	C	T	0.252874	0.0266954	0.71506707
chr6	150240829	RAET1G	rs9397449	G	C	0.12069	0.0267337	0.71506707
chr19	37854580	HKR1	rs3745764	C	T	0.252874	0.026741	0.71506707
chr14	77493791	IRF2BPL	rs773628724	CTGT	C	0.494253	0.0268427	0.71506707
chr1	27278573	C1orf172	rs17360994	T	C	0.0804598	0.0268641	0.71506707
chr1	27239920	NR0B2	rs6659176	C	G	0.0804598	0.0268641	0.71506707
chr19	52249211	FPR1	rs867228	T	G	0.804598	0.0268872	0.71506707
chr14	105414238	AHNAK2	rs60754080	C	A	0.482759	0.0269601	0.71506707

chr12	5841733	ANO2	rs1860961	A	C	0.0804598	0.0269655	0.71506707
chr15	91543761	VPS33B	rs11073964	C	T	0.511494	0.0269738	0.71506707
chr14	24458211	DHRS4L2	rs2273947	A	T	0.091954	0.0269759	0.71506707
chr22	19119751	DGCR14	rs1052763	C	T	0.16092	0.0270556	0.71506707
chr22	19119751	TSSK2	rs1052763	C	T	0.16092	0.0270556	0.71506707
chr6	116600774	TSPYL1	rs3749895	C	G	0.264368	0.0271428	0.71506707
chr9	35606884	TESK1	rs2275422	G	A	0.482759	0.0272307	0.71506707
chr2	30980940	CAPN13	rs508405	C	T	0.678161	0.0272889	0.71506707
chr2	74692191	MOGS	rs79181168	C	T	0.0632184	0.0272946	0.71506707
chr11	75378674	MAP6	rs12225010	A	C	0.591954	0.027302	0.71506707
chr3	99886662	C3orf26	rs11537816	G	A	0.218391	0.0273161	0.71506707
chr18	60383787	PHLPP1	rs187210763	T	G	0.0574713	0.0273932	0.71506707
chr18	60383982	PHLPP1	rs12957017	G	A	0.0574713	0.0273932	0.71506707
chr19	9024994	MUC16	rs67631215	C	T	0.327586	0.0274067	0.71506707
chr18	47908556	SKA1	rs6507992	G	A	0.637931	0.0274988	0.71506707
chr1	149906413	MTMR11	rs11205303	T	C	0.390805	0.027527	0.71506707
chr16	58019396	TEPP	rs9934227	G	A	0.816092	0.0275474	0.71506707
chr20	36932660	BPI	rs1341023	C	T	0.557471	0.0275847	0.71506707
chr2	55404794	C2orf63	rs14026	G	A	0.534483	0.0276096	0.71506707
chr7	123256427	ASB15	rs6962756	C	T	0.672414	0.0276705	0.71506707
chr1	175375469	TNR	rs2239819	C	A	0.293103	0.027696	0.71506707
chr22	32871383	FBXO7	rs8137714	T	G	0.241379	0.0277422	0.71506707
chr7	50173777	C7orf72	rs998928	A	G	0.396552	0.0277429	0.71506707
chr11	102272884	TMEM123	rs2155587	C	T	0.109195	0.0277704	0.71506707
chr7	144094613	NOBOX	rs1208216	G	T	0.143678	0.0278295	0.71506707
chr7	144096068	NOBOX	rs2525702	C	T	0.143678	0.0278295	0.71506707
chr5	122435627	PRDM6	rs1008058	G	A	0.0804598	0.0279258	0.71506707
chr4	187629770	FAT1	rs3733414	A	C	0.609195	0.0279642	0.71506707
chr1	179989742	CEP350	rs2477120	G	C	0.62069	0.0280108	0.71506707
chr10	103920475	NOLC1	rs1049455	T	C	0.114943	0.0280902	0.71506707
chr20	2559795	TMC2	rs34884202	T	C	0.0689655	0.0281019	0.71506707

chr6	112671611	RFPL4B	rs11153361	G	A	0.229885	0.0281454	0.71506707
chr2	178482594	TTC30A	rs61742858	G	T	0.132184	0.0281584	0.71506707
chr17	72308319	DNAI2	rs1979370	G	A	0.91954	0.0281939	0.71506707
chr1	19228971	ALDH4A1	rs146450609	G	A	0.0574713	0.0281995	0.71506707
chr5	140574169	PCDHB10	rs140613424	CAGGCCG	C	0.344828	0.0282517	0.71506707
chr4	110901198	EGF	rs2237051	G	A	0.396552	0.028336	0.71506707
chr19	14141666	RLN3	rs78161395	G	T	0.132184	0.0283392	0.71506707
chr1	98165091	DPYD	rs2297595	T	C	0.0517241	0.0284541	0.71506707
chrX	48418126	TBC1D25	rs2293948	A	G	0.304598	0.0284761	0.71506707
chrX	48418659	TBC1D25	rs235836	G	A	0.304598	0.0284761	0.71506707
chr19	53667804	ZNF665	rs4801958	C	T	0.637931	0.0285	0.71506707
chr19	53669492	ZNF665	rs12460170	A	G	0.637931	0.0285	0.71506707
chr11	5701074	TRIM5	rs11601507	C	A	0.0804598	0.0285514	0.71506707
chr12	21011480	SLCO1B3	rs4149117	T	G	0.890805	0.0285709	0.71506707
chr12	21015760	SLCO1B3	rs7311358	G	A	0.890805	0.0285709	0.71506707
chr2	160676427	LY75	rs12692566	C	A	0.764368	0.028691	0.71506707
chr2	160676427	LY75-CD302	rs12692566	C	A	0.764368	0.028691	0.71506707
chr10	74894375	ECD	rs2271904	T	C	0.0689655	0.0287039	0.71506707
chr10	74896664	ECD	rs36152134	T	C	0.0689655	0.0287039	0.71506707
chrX	1531687	ASMTL	rs79968027	T	C	0.0747126	0.0288162	0.71506707
chrX	1531687	ASMTL-AS1	rs79968027	T	C	0.0747126	0.0288162	0.71506707
chr19	57649900	ZIM3	rs2370134	C	T	0.178161	0.028834	0.71506707
chr12	95681597	VEZT	rs10507051	G	A	0.0632184	0.0288453	0.71506707
chr12	52639302	KRT7	rs2608009	G	C	0.83908	0.0288677	0.71506707
chr11	67809268	TCIRG1	rs36027301	C	T	0.0689655	0.0289059	0.71506707
chr2	95537622	TEKT4	rs11164112	A	G	0.413793	0.0289446	0.71506707
chr7	36656035	AOAH	rs3735386	G	C	0.109195	0.0290071	0.71506707
chr10	129905896	MKI67	rs3740423	T	A	0.189655	0.0290316	0.71506707
chr5	34840841	TTC23L	rs6451173	A	G	0.5	0.0290569	0.71506707
chr13	111119396	COL4A2	rs3803230	G	C	0.0747126	0.0290904	0.71506707
chr18	66542006	CCDC102B	rs745894	G	T	0.195402	0.0291494	0.71506707

chr19	48800914	CCDC114	rs35461177	G	A	0.270115	0.0292341	0.71506707
chr1	207074905	IL24	rs1150258	T	C	0.436782	0.0292402	0.71506707
chr10	5138747	AKR1C3	rs11551177	A	G	0.0574713	0.0292418	0.71506707
chr21	43824106	UBASH3A	rs2277798	A	G	0.58046	0.0293762	0.71506707
chr15	91326099	BLM	rs2227935	C	T	0.0689655	0.0294313	0.71506707
chr15	91354521	BLM	rs7167216	G	A	0.0689655	0.0294313	0.71506707
chr6	30993776	PBMUCL1	rs115709409	A	T	0.114943	0.0294336	0.71506707
chr1	14105139	PRDM2	rs2076324	T	A	0.293103	0.0294696	0.71506707
chr16	88779739	CTU2	rs11549837	A	G	0.356322	0.0294882	0.71506707
chr5	140580931	PCDHB11	rs799834	C	G	0.41954	0.0294973	0.71506707
chr5	140559532	PCDHB8	rs2740582	G	C	0.413793	0.0295508	0.71506707
chr9	117846580	TNC	rs1061494	T	C	0.402299	0.0296104	0.71506707
chr16	67964203	CTRL	rs1134760	T	C	0.143678	0.0297491	0.71506707
chr1	228558892	OBSCN	rs35186354	C	T	0.103448	0.0297987	0.71506707
chr8	70980738	PRDM14	rs3750228	T	C	0.149425	0.0298026	0.71506707
chr11	68678962	IGHMBP2	rs560096	T	C	0.867816	0.0298495	0.71506707
chr5	475104	SLC9A3	rs2247114	A	G	0.850575	0.0299125	0.71506707
chr17	53076799	STXBP4	rs1156287	G	A	0.793103	0.0299607	0.71506707
chr12	88380094	C12orf50	rs10777084	T	C	0.103448	0.0300068	0.71506707
chr4	2951804	NOP14	rs2515960	A	G	0.344828	0.030069	0.71506707
chr18	11689669	GNAL	rs201898548	C	CGGCCCT	0.178161	0.0300815	0.71506707
chr7	149486294	SSPO	rs855691	A	G	0.0747126	0.0301071	0.71506707
chr7	149486382	SSPO	rs2074704	C	G	0.0747126	0.0301071	0.71506707
chr7	149489491	SSPO	rs1076277	C	T	0.0747126	0.0301071	0.71506707
chr11	58170792	OR5B3	rs200799158	AG	A	0.0632184	0.0301355	0.71506707
chr20	46279836	NCOA3	rs147879509	ACAG	A	0.321839	0.0302842	0.71506707
chr10	7749183	ITIH2	rs73621253	C	T	0.0977011	0.0303033	0.71506707
chr22	22989256	GGTLC2	rs2904923	A	G	0.867816	0.0303129	0.71506707
chr1	169511555	F5	rs6032	T	C	0.270115	0.0303806	0.71506707
chr1	169511734	F5	rs4525	T	C	0.270115	0.0303806	0.71506707
chr1	169511755	F5	rs4524	T	C	0.270115	0.0303806	0.71506707

chr5	140590766	PCDHB12	rs2910006	A	G	0.362069	0.030418	0.71506707
chr5	140595106	PCDHB13	rs2910329	C	G	0.362069	0.030418	0.71506707
chr2	242011084	SNED1	rs17440466	T	C	0.0747126	0.0304453	0.71506707
chr19	44471209	ZNF221	rs365745	T	A	0.798851	0.0304814	0.71506707
chr19	18375882	KIAA1683	rs12608777	G	C	0.149425	0.0307347	0.71570947
chr19	18377761	KIAA1683	rs12609001	A	G	0.149425	0.0307347	0.71570947
chr17	39577215	KRT37	rs8071814	G	A	0.0862069	0.0308164	0.71570947
chr11	56468720	OR9G9	rs66943455	T	C	0.482759	0.0308261	0.71570947
chr11	56468720	OR9G1	rs66943455	T	C	0.482759	0.0308261	0.71570947
chr10	126172863	LHPP	rs6597801	A	G	0.862069	0.0308392	0.71570947
chr1	16727305	SPATA21	rs139527123	G	GCTT	0.149425	0.0308998	0.71570947
chr22	43579083	TTLL12	rs138951	G	A	0.103448	0.0309133	0.71570947
chr11	56185159	OR5R1	rs7930678	A	G	0.206897	0.030966	0.71570947
chr11	56185327	OR5R1	rs7933772	T	C	0.206897	0.030966	0.71570947
chr11	56185689	OR5R1	rs7931261	A	G	0.206897	0.030966	0.71570947
chr12	129189941	TMEM132C	rs12424159	G	A	0.626437	0.0310883	0.7160279
chr15	30018627	TJP1	rs2229515	T	C	0.114943	0.0310949	0.7160279
chr1	151881885	THEM4	rs3748805	A	C	0.873563	0.0311144	0.7160279
chr19	10273372	DNMT1	rs2228612	T	C	0.091954	0.0311986	0.7160279
chr14	105416872	AHNAK2	rs148787429	T	C	0.0574713	0.0312402	0.7160279
chr6	29274486	OR14J1	rs9257694	T	C	0.505747	0.0312556	0.7160279
chr17	15234895	TEKT3	rs7226363	C	T	0.224138	0.0313044	0.7160279
chr19	42085873	CEACAM21	rs2302188	A	G	0.747126	0.0313266	0.7160279
chr14	105410411	AHNAK2	rs11160825	C	T	0.494253	0.0314489	0.7160279
chr6	29407970	OR10C1	rs2074469	T	C	0.201149	0.031476	0.7160279
chr11	8662516	TRIM66	rs11042023	T	C	0.614943	0.0315623	0.7160279
chr16	29908433	SEZ6L2	rs11649499	C	G	0.83908	0.0315872	0.7160279
chr12	6711147	CHD4	rs1639122	C	A	0.413793	0.0317151	0.7160279
chr4	178256913	NEIL3	rs7689099	C	G	0.12069	0.0317278	0.7160279
chr2	216923679	PECR	rs1429148	C	T	0.0804598	0.0317304	0.7160279
chr16	89180883	ACSF3	rs3743979	G	A	0.718391	0.0317564	0.7160279

chr17	79986156	LRRC45	rs72861736	C	T	0.0862069	0.0317921	0.7160279
chr5	179740827	GFPT2	rs2303007	T	C	0.206897	0.0318342	0.7160279
chr22	31621792	LIMK2	rs5997917	G	A	0.166667	0.0318396	0.7160279
chr1	3413868	MEGF6	rs4648506	C	G	0.252874	0.031844	0.7160279
chr13	25075864	PARP4	rs35200240	T	C	0.0804598	0.0319119	0.7160279
chr16	77359919	ADAMTS18	rs11640912	A	T	0.402299	0.0319309	0.7160279
chr5	140778256	PCDHGB5	rs6867460	G	A	0.143678	0.0319362	0.7160279
chr9	94486321	ROR2	rs10761129	C	T	0.735632	0.0319938	0.71638653
chr1	228504591	OBSCN	rs61825301	C	A	0.149425	0.0322524	0.71872683
chr1	228505739	OBSCN	rs3795801	G	A	0.149425	0.0322524	0.71872683
chr6	29012067	LOC100129636	rs35771565	C	T	0.172414	0.0323545	0.71872683
chr6	29012067	OR2W1	rs35771565	C	T	0.172414	0.0323545	0.71872683
chr6	29080349	OR2J3	rs3130764	A	G	0.827586	0.0323638	0.71872683
chr16	633353	PIGQ	rs710924	T	C	0.362069	0.0323905	0.71872683
chr16	633354	PIGQ	rs710925	G	A	0.362069	0.0323905	0.71872683
chr1	50666515	ELAVL4	rs2494876	C	T	0.936782	0.0324674	0.719506
chr4	187629538	FAT1	rs3733413	C	T	0.545977	0.032517	0.71967895
chr18	5890571	TMEM200C	rs7506026	T	C	0.0977011	0.0326276	0.7197564
chr15	89836228	FANCI	rs2283432	G	C	0.350575	0.0326858	0.7197564
chr5	79734297	ZFYVE16	rs259028	T	C	0.91954	0.0326877	0.7197564
chr5	79745469	ZFYVE16	rs249038	A	G	0.91954	0.0326877	0.7197564
chr3	118621500	IGSF11	rs34908332	C	T	0.109195	0.0327779	0.72018468
chr4	2943361	NOP14	rs1054090	T	C	0.333333	0.0327908	0.72018468
chr14	105413204	AHNAK2	rs2582514	G	T	0.494253	0.0329845	0.72203796
chr17	15496727	CDRT1	rs79385100	T	G	0.126437	0.0330048	0.72203796
chr5	132652281	FSTL4	rs17683306	C	T	0.109195	0.0331632	0.72203796
chr10	95381773	PDE6C	rs701865	T	A	0.344828	0.0332473	0.72203796
chr3	97983487	OR5H6	rs398062605	TTGTAACCAC	T	0.557471	0.0332514	0.72203796
chr3	97983981	OR5H6	rs9871143	G	A	0.557471	0.0332514	0.72203796
chr14	24587639	DCAF11	rs3825584	G	A	0.298851	0.0332659	0.72203796

chr12	109017898	SELPLG	rs2228315	C	T	0.0862069	0.0333748	0.72203796
chr20	210306	DEFB129	rs1053783	C	G	0.155172	0.0333759	0.72203796
chr15	45444133	DUOX1	rs16939752	T	C	0.12069	0.0335349	0.72203796
chr6	27925367	OR2B6	rs7767176	G	A	0.0977011	0.0335916	0.72203796
chr1	247875415	OR6F1	rs2282316	A	G	0.218391	0.0335954	0.72203796
chr3	93780109	DHFRL1	rs61739170	G	C	0.201149	0.033601	0.72203796
chr12	48272895	VDR	rs2228570	A	G	0.66092	0.0336298	0.72203796
chr11	55606693	OR5D16	rs6591700	G	A	0.0862069	0.0336385	0.72203796
chr17	18671961	FBXW10	rs79757327	C	T	0.0632184	0.0336752	0.72203796
chr22	30856121	SEC14L3	rs35764129	G	A	0.0517241	0.0337038	0.72203796
chr11	47857253	NUP160	rs3816605	T	C	0.465517	0.0337062	0.72203796
chr4	95170839	SMARCAD1	rs11722476	G	A	0.37931	0.0337688	0.72203796
chr19	38375666	WDR87	rs73027451	C	T	0.132184	0.033797	0.72203796
chr17	9515777	WDR16	rs6503235	G	A	0.735632	0.0337977	0.72203796
chr17	60814273	MARCH10	rs9891498	A	G	0.408046	0.0338492	0.7222421
chr2	20870820	GDF7	rs78872016	G	T	0.0862069	0.0339098	0.72252412
chr19	10114252	COL5A3	rs61742765	C	G	0.212644	0.0339883	0.72252412
chr19	10114740	COL5A3	rs62104337	T	A	0.212644	0.0339883	0.72252412
chr12	86373221	MGAT4C	rs17855890	G	C	0.0977011	0.034188	0.72587321
chr9	127177161	PSMB7	rs4574	A	G	0.448276	0.0342734	0.72647583
chr17	76503593	DNAH17	rs9896398	T	C	0.597701	0.0343429	0.72647583
chr17	3657159	ITGAE	rs2272606	C	T	0.758621	0.0344045	0.72647583
chr20	61513649	DIDO1	rs6011441	G	T	0.114943	0.0345332	0.72647583
chr17	56435080	RNF43	rs9652855	G	C	0.126437	0.0345484	0.72647583
chr16	701656	WDR90	rs11642546	C	T	0.218391	0.0345935	0.72647583
chr16	2160973	PKD1	rs116092985	A	G	0.132184	0.0345951	0.72647583
chr16	2162361	PKD1	rs2549677	A	G	0.132184	0.0345951	0.72647583
chr21	43896143	RSPH1	rs117385282	C	T	0.201149	0.0346152	0.72647583
chr2	228163453	COL4A3	rs57611801	C	A	0.0517241	0.0347682	0.72647583
chr3	108298260	KIAA1524	rs2278911	C	T	0.103448	0.0347798	0.72647583
chr15	78390414	SH2D7	rs2289524	T	C	0.41954	0.034798	0.72647583

chr11	62863518	SLC22A24	rs7113279	A	G	0.603448	0.0348563	0.72647583
chr1	6533393	PLEKHG5	rs61741379	G	C	0.137931	0.0349112	0.72647583
chr1	6184092	CHD5	rs2843493	A	G	0.62069	0.0349127	0.72647583
chr6	160952838	LPA	rs3124784	G	A	0.281609	0.0349331	0.72647583
chr16	88599659	ZFPM1	rs71395304	G	T	0.12069	0.0349822	0.72647583
chr22	23503121	RAB36	rs5759611	G	A	0.247126	0.0349977	0.72647583
chr4	38774889	TLR10	rs4129009	T	C	0.126437	0.035018	0.72647583
chr1	240255568	FMN2	rs140531536	GGGC	G	0.781609	0.0352093	0.72951507
chr22	20103263	TRMT2A	rs9605067	G	T	0.0517241	0.0352579	0.72951507
chr1	42047208	HIVEP3	rs17363472	C	G	0.0689655	0.0353516	0.72951507
chr14	105055118	C14orf180	rs111285011	TGACGGGCAG	T	0.465517	0.035357	0.72951507
chr18	61602360	SERPINB10	rs35453062	G	A	0.109195	0.0353798	0.72951507
chr2	242046785	PASK	rs1131293	A	C	0.0747126	0.0354187	0.72951507
chr19	43680037	PSG5	rs1135901	T	A	0.172414	0.035531	0.73008149
chr19	43680269	PSG5	rs1058259	G	C	0.172414	0.035531	0.73008149
chr7	21775369	DNAH11	rs68023059	G	A	0.0574713	0.0356353	0.73064865
chr15	63597857	APH1B	rs1047552	T	G	0.0689655	0.035652	0.73064865
chr14	24845841	NFATC4	rs7149586	T	C	0.701149	0.0356859	0.73064865
chr8	90784979	RIPK2	rs2230801	T	C	0.0574713	0.0357697	0.73149461
chr15	93015427	C15orf32	rs1455773	G	A	0.298851	0.035837	0.73152425
chr19	29704010	UQCRFS1	rs8100724	A	C	0.896552	0.035887	0.73152425
chr19	45655647	NKPD1	rs117934605	G	T	0.114943	0.0358986	0.73152425
chr9	113259152	SVEP1	rs10817033	T	G	0.109195	0.0360575	0.73272024
chr11	66033430	KLC2	rs2276036	C	T	0.178161	0.0361019	0.73272024
chr10	133795391	BNIP3	rs11556626	A	C	0.126437	0.036133	0.73272024
chr1	27332466	FAM46B	rs61737590	T	C	0.0747126	0.0362352	0.73272024
chr16	3339435	ZNF263	rs220379	G	C	0.218391	0.0362941	0.73272024
chr19	58355699	LOC100293516	rs8103104	T	C	0.396552	0.036331	0.73272024
chr6	168442765	KIF25	rs2073634	G	A	0.103448	0.0363475	0.73272024
chr5	140739779	PCDHGB2	rs17097231	C	G	0.12069	0.0363813	0.73272024

chr2	73716810	ALMS1	rs3820700	G	A	0.0977011	0.0364879	0.73272024
chr2	73717103	ALMS1	rs2017116	G	C	0.0977011	0.0364879	0.73272024
chr2	73675669	ALMS1	rs2037814	T	G	0.902299	0.0364997	0.73272024
chr13	28552425	PRHOXNB	rs9579139	G	T	0.333333	0.0365656	0.73272024
chr7	149523195	SSPO	rs78492471	C	G	0.091954	0.0366101	0.73272024
chr3	98073591	OR5K4	rs11288615	TA	T	0.563218	0.0366539	0.73272024
chr1	47016761	KNCN	rs61997191	C	T	0.0517241	0.0366995	0.73272024
chr4	88583135	DMP1	rs10019009	A	T	0.247126	0.0367183	0.73272024
chr14	35735967	KIAA0391	rs11156878	A	G	0.172414	0.0368318	0.73272024
chr22	21354970	THAP7	rs426938	C	G	0.798851	0.036832	0.73272024
chr18	33831189	MOCOS	rs594445	C	A	0.275862	0.0368795	0.73272024
chr4	1843324	LETM1	rs116753949	C	T	0.103448	0.0369127	0.73272024
chr20	44520237	CTSA	rs540291608	CCTG	C	0.637931	0.0369388	0.73272024
chr20	44520237	CTSA	rs397784956	CCTG	C	0.637931	0.0369388	0.73272024
chr2	37406680	SULT6B1	rs10205833	C	G	0.241379	0.0369661	0.73272024
chr14	76156609	TTLL5	rs2303345	C	T	0.643678	0.0370474	0.73272024
chr9	75315438	TMC1	rs1796993	G	A	0.183908	0.0371071	0.73272024
chr12	108618630	WSCD2	rs3764002	C	T	0.270115	0.0371637	0.73272024
chr19	50093248	PRRG2	rs144521999	A	T	0.126437	0.0372063	0.73272024
chr1	223954080	CAPN2	rs17599	A	C	0.218391	0.0372079	0.73272024
chr20	31619500	BPIL3	rs17301126	C	T	0.103448	0.0372196	0.73272024
chr4	153896358	FHDC1	rs3811833	C	T	0.741379	0.0372401	0.73272024
chr10	98924600	ARHGAP19-SLIT1	rs41307074	G	A	0.0574713	0.0373293	0.73272024
chr10	98924600	SLIT1	rs41307074	G	A	0.0574713	0.0373293	0.73272024
chrX	138633280	F9	rs6048	A	G	0.143678	0.0373604	0.73272024
chr22	28194933	MN1	rs45480998	T	TTGC	0.16092	0.0373748	0.73272024
chr22	28194933	MN1	rs34890218	T	TTGC	0.16092	0.0373748	0.73272024
chr22	30762140	CCDC157	rs740223	G	A	0.258621	0.0374864	0.73272024
chr22	30776095	RNF215	rs5749088	C	T	0.258621	0.0374864	0.73272024
chr22	44368122	SAMM50	rs3761472	A	G	0.132184	0.0374892	0.73272024

chrX	150817094	PASD1	rs5924658	C	G	0.149425	0.0375484	0.73304524
chr22	38877461	KDELR3	rs12004	T	G	0.304598	0.0376007	0.73323494
chr5	93987532	ANKRD32	rs6891545	C	A	0.178161	0.0377827	0.73360061
chr16	1252369	CACNA1H	rs61734410	C	T	0.316092	0.037799	0.73360061
chr17	39137297	KRT40	rs721957	C	T	0.58046	0.0378099	0.73360061
chr13	44411432	CCDC122	rs9567280	A	G	0.0747126	0.0382236	0.73360061
chr5	79616936	SPZ1	rs35337118	A	C	0.178161	0.0382761	0.73360061
chr19	12186921	ZNF844	rs8102258	T	C	0.0804598	0.0382871	0.73360061
chr9	98638288	C9orf102	rs690528	A	G	0.373563	0.0383	0.73360061
chr13	29855847	MTUS2	rs3751336	C	T	0.0574713	0.0384386	0.73360061
chr4	48082095	TXK	rs11724347	C	T	0.0517241	0.0384414	0.73360061
chr5	140562739	PCDHB16	rs61743469	G	A	0.0689655	0.0384554	0.73360061
chr12	10782115	STYK1	rs3759259	T	C	0.54023	0.0384621	0.73360061
chr20	57829301	ZNF831	rs259956	T	C	0.505747	0.0385659	0.73360061
chr4	5894398	CRMP1	rs139357095	C	G	0.0517241	0.038571	0.73360061
chr8	30703431	TEX15	rs323346	T	C	0.149425	0.0385997	0.73360061
chr8	30706224	TEX15	rs323347	A	G	0.149425	0.0385997	0.73360061
chr6	30558477	ABCF1	rs4148252	G	GA	0.701149	0.0386127	0.73360061
chr7	6193752	USP42	rs61753119	G	A	0.114943	0.0386133	0.73360061
chr16	70934974	HYDIN	rs12102425	T	C	0.0689655	0.0386441	0.73360061
chr20	62194713	PRIC285	rs3810486	A	C	0.212644	0.0387788	0.73360061
chr20	62195220	PRIC285	rs3827022	C	T	0.212644	0.0387788	0.73360061
chr20	62196033	PRIC285	rs3810487	C	T	0.212644	0.0387788	0.73360061
chr20	62200576	PRIC285	rs6090457	G	A	0.212644	0.0387788	0.73360061
chr7	150325175	GIMAP6	rs13234724	C	T	0.201149	0.0388072	0.73360061
chr6	49814379	CRISP1	rs3209304	T	C	0.126437	0.0388142	0.73360061
chr4	104066461	CENPE	rs2615542	A	G	0.183908	0.0388535	0.73360061
chr6	32549588	HLA-DRB1	rs201929247	G	A	0.212644	0.0388809	0.73360061
chr17	41931375	CD300LG	rs12453522	A	G	0.218391	0.0389301	0.73360061
chr16	3254470	OR1F1	rs1834026	T	C	0.494253	0.0389734	0.73360061
chr1	24421474	MYOM3	rs6678540	G	A	0.396552	0.0390068	0.73360061

chr14	88852166	SPATA7	rs4904448	G	A	0.385057	0.0391082	0.73360061
chr2	160729005	LY75-CD302	rs1397706	C	T	0.0747126	0.0391151	0.73360061
chr2	160729005	LY75	rs1397706	C	T	0.0747126	0.0391151	0.73360061
chr3	157081324	VEPH1	rs11918974	A	G	0.287356	0.0391891	0.73360061
chr4	57215677	AASDH	rs3796544	G	A	0.454023	0.0392828	0.73360061
chr16	19041595	TMC7	rs4072393	G	A	0.666667	0.0394334	0.73360061
chr10	77161100	ZNF503	.	G	GCCGCCTCCG CCT	0.0632184	0.0395929	0.73360061
chr10	77161100	ZNF503	rs762649774	G	GCCGCCTCCG CCT	0.0632184	0.0395929	0.73360061
chr14	105408182	AHNAK2	rs9672139	T	G	0.482759	0.0396349	0.73360061
chr19	1397443	GAMT	rs17851582	G	A	0.0804598	0.0396541	0.73360061
chr11	65735174	SART1	rs660118	G	C	0.396552	0.0396849	0.73360061
chr1	153004853	SPRR1B	rs3795382	C	T	0.511494	0.0397086	0.73360061
chr2	68385097	PNO1	rs2044693	A	G	0.655172	0.0397168	0.73360061
chr2	47382342	C2orf61	rs815804	G	T	0.178161	0.0397229	0.73360061
chr5	38955796	RICTOR	rs2043112	G	A	0.442529	0.0397831	0.73360061
chr16	71683718	PHLPP2	rs61733127	A	G	0.183908	0.0399007	0.73360061
chr4	40778162	NSUN7	rs2437323	T	G	0.804598	0.0399037	0.73360061
chr8	17396380	SLC7A2	rs13259948	G	A	0.183908	0.0399143	0.73360061
chr18	48346024	MRO	rs3813089	G	T	0.224138	0.0399365	0.73360061
chr10	63977980	RTKN2	rs61850830	C	T	0.0862069	0.0399436	0.73360061
chr5	68616331	CCDC125	rs10471774	C	T	0.471264	0.0399699	0.73360061
chr6	41903782	CCND3	rs1051130	A	C	0.517241	0.0400044	0.73360061
chr1	3679775	CCDC27	rs10910024	C	T	0.235632	0.0400553	0.73360061
chr20	44004155	TP53TG5	rs10546815	CATT	C	0.672414	0.0400819	0.73360061
chr20	44004155	SYS1	rs10546815	CATT	C	0.672414	0.0400819	0.73360061
chr15	99762041	TTC23	rs78534478	C	T	0.0689655	0.040084	0.73360061
chr4	71468345	AMBN	rs141384720	CAGG	C	0.091954	0.0400875	0.73360061
chr4	71469604	AMBN	rs7439186	C	T	0.091954	0.0400875	0.73360061
chr20	34596371	C20orf152	rs6060750	C	T	0.178161	0.0401375	0.73360061
chr10	103298099	BTRC	rs4151060	G	T	0.0804598	0.0401404	0.73360061

chr5	168212951	SLIT3	rs891921	A	G	0.902299	0.0401511	0.73360061
chr10	72015573	NPFFR1	rs3812694	T	G	0.0804598	0.0401757	0.73360061
chr14	100705787	YY1	rs568477380	GCCA	G	0.0517241	0.0403222	0.73473017
chr21	46641968	ADARB1	rs1051367	G	A	0.517241	0.0403229	0.73473017
chr2	61483548	USP34	rs72811482	A	T	0.0804598	0.040428	0.73586652
chr1	15909744	AGMAT	rs11580170	C	T	0.247126	0.040816	0.74002597
chr15	85163998	ZSCAN2	rs2044502	G	C	0.850575	0.0408881	0.74002597
chr7	141752215	MGAM	rs116536012	A	C	0.0574713	0.0408972	0.74002597
chr8	145541766	DGAT1	rs55962377	G	T	0.0747126	0.0408983	0.74002597
chr15	84651057	ADAMTSL3	rs140206840	G	A	0.0574713	0.0410044	0.74002597
chr5	149755744	TCOF1	rs2071240	G	C	0.0632184	0.0410927	0.74002597
chr19	17337882	OCEL1	rs891203	C	G	0.137931	0.041111	0.74002597
chr10	71060610	HK1	rs906220	A	G	0.885057	0.0411927	0.74002597
chr15	102264304	TARSL2	rs1143138	G	C	0.304598	0.0413026	0.74002597
chr14	45606287	FANCM	rs10138997	C	T	0.0574713	0.041446	0.74002597
chr9	100122291	C9orf174	rs3747495	T	C	0.143678	0.0414499	0.74002597
chr9	100122291	BDAG1	rs3747495	T	C	0.143678	0.0414499	0.74002597
chr11	56185345	OR5R1	rs6591324	A	G	0.758621	0.0414715	0.74002597
chr10	115804036	ADRB1	rs1801252	A	G	0.143678	0.0415369	0.74002597
chr6	165703523	C6orf118	rs17856754	C	T	0.0747126	0.0415662	0.74002597
chr6	165706935	C6orf118	rs9459350	C	G	0.0747126	0.0415662	0.74002597
chr19	55349273	KIR2DS4	rs1130492	C	A	0.143678	0.0418135	0.74002597
chrX	1314931	CRLF2	rs151218732	C	T	0.0862069	0.0418435	0.74002597
chr16	1306346	TPSD1	rs3865205	C	T	0.0804598	0.0418523	0.74002597
chr20	62373707	SLC2A4RG	rs8957	G	T	0.781609	0.0420256	0.74002597
chr15	42681199	CAPN3	rs1801449	G	A	0.0517241	0.0421107	0.74002597
chr14	105965102	C14orf80	rs34137879	G	A	0.0517241	0.0421249	0.74002597
chr19	51361757	KLK3	rs17632542	T	C	0.0977011	0.0421502	0.74002597
chr2	48921375	LHCGR	rs2293275	T	C	0.649425	0.0422548	0.74002597
chr12	52284500	ANKRD33	rs34494292	A	G	0.281609	0.0422894	0.74002597
chr12	52285086	ANKRD33	rs3180417	G	A	0.281609	0.0422894	0.74002597

chr17	39383073	KRTAP9-2	rs9902235	G	C	0.66092	0.0422948	0.74002597
chr3	196388099	LRRC33	rs62623661	G	C	0.137931	0.0422988	0.74002597
chr5	37173930	C5orf42	rs10076911	A	C	0.0689655	0.0423151	0.74002597
chr5	37182902	C5orf42	rs75589774	G	A	0.0689655	0.0423151	0.74002597
chr16	54318172	IRX3	rs1126960	C	A	0.247126	0.0424369	0.74002597
chr2	73675227	ALMS1	rs34628045	T	TCTC	0.632184	0.042447	0.74002597
chr2	73828538	ALMS1	rs1052161	G	A	0.632184	0.042447	0.74002597
chr19	8670147	ADAMTS10	rs62621197	C	T	0.0517241	0.0424709	0.74002597
chr19	54782919	LILRB2	rs386056	C	T	0.195402	0.0424764	0.74002597
chr19	54784130	LILRB2	rs383369	T	C	0.195402	0.0424764	0.74002597
chr21	43413553	ZNF295	rs871546	T	G	0.258621	0.0425447	0.74002597
chr7	21940823	CDCA7L	rs77448980	A	G	0.0747126	0.0426057	0.74002597
chr7	21940823	DNAH11	rs77448980	A	G	0.0747126	0.0426057	0.74002597
chr1	117529458	PTGFRN	rs10801922	G	A	0.37931	0.0426461	0.74002597
chr19	52249947	FPR1	rs2070745	C	G	0.310345	0.0426526	0.74002597
chr8	87076520	PSKH2	rs6998760	C	A	0.321839	0.0426863	0.74002597
chr17	76134237	TMC8	rs11651675	G	A	0.0517241	0.0426914	0.74002597
chr15	40558064	PAK6	rs2412504	A	G	0.0977011	0.0427087	0.74002597
chr15	40558482	PAK6	rs3743135	A	G	0.0977011	0.0427087	0.74002597
chr17	30183857	C17orf79	rs8068049	T	C	0.833333	0.0427493	0.74002597
chr2	218713282	TNS1	rs3796033	G	A	0.373563	0.0427601	0.74002597
chr1	110279701	GSTM3	rs7483	C	T	0.316092	0.0427804	0.74002597
chr6	133045902	VNN3	rs2294759	A	G	0.287356	0.042802	0.74002597
chr5	13902220	DNAH5	rs1530498	T	C	0.350575	0.0428292	0.74002597
chr2	207631461	FASTKD2	rs3762568	G	A	0.0574713	0.0429287	0.74002597
chr19	8138054	FBN3	rs7257948	C	A	0.568966	0.0429386	0.74002597
chr19	56001665	SSC5D	rs114976626	C	T	0.0517241	0.0430563	0.74002597
chr19	56011573	SSC5D	rs61747393	C	T	0.0517241	0.0430563	0.74002597
chr19	55993436	ZNF628	rs147110934	G	T	0.0517241	0.0430563	0.74002597
chr12	93196422	EEA1	rs10745623	T	G	0.586207	0.0430776	0.74002597
chr11	6555365	DNHD1	rs7103810	A	G	0.885057	0.0431278	0.74002597

chr13	50141345	RCBTB1	rs4942848	G	A	0.66092	0.0431492	0.74002597
chr3	124746049	HEG1	rs2981546	A	G	0.5	0.0432788	0.74123687
chr4	169362557	DDX60L	rs13151700	C	G	0.373563	0.0433059	0.74123687
chr14	70924602	ADAM21	rs72735759	T	G	0.0574713	0.0434196	0.74170843
chr14	70925501	ADAM21	rs72735760	G	C	0.0574713	0.0434196	0.74170843
chr1	24447468	IL22RA1	rs3795299	G	C	0.333333	0.0434788	0.7419836
chr10	124742895	PSTK	rs3736582	G	C	0.655172	0.0436983	0.7449552
chr3	124732618	HEG1	rs6790837	A	G	0.505747	0.0438311	0.7449552
chr7	106509331	PIK3CG	rs17847825	C	A	0.0862069	0.0438729	0.7449552
chr19	3831449	ZFR2	rs2240232	G	A	0.362069	0.0439928	0.7449552
chr19	3831525	ZFR2	rs2240233	C	A	0.362069	0.0439928	0.7449552
chr19	3831765	ZFR2	rs2240235	G	A	0.362069	0.0439928	0.7449552
chr1	67243024	TCTEX1D1	rs2133173	C	A	0.327586	0.0440068	0.7449552
chr16	20810067	ERI2	rs3213646	T	C	0.534483	0.0441801	0.7449552
chr1	79095581	IFI44L	rs987495	T	C	0.195402	0.0443096	0.7449552
chr1	109391662	AKNAD1	rs11580913	G	C	0.0977011	0.0443222	0.7449552
chr19	11979305	ZNF439	rs72994214	C	G	0.0574713	0.0444337	0.7449552
chr12	21201663	LST-3TM12	rs11045689	G	A	0.689655	0.0444693	0.7449552
chr1	100598866	CCDC76	rs472498	G	A	0.873563	0.0444859	0.7449552
chr1	100598867	CCDC76	rs687513	C	T	0.873563	0.0444859	0.7449552
chr4	1019011	FGFRL1	rs4647932	C	T	0.0574713	0.0445608	0.7449552
chr17	56598991	SEPT4	rs17741424	T	A	0.149425	0.0445972	0.7449552
chr20	62326110	RTEL1-TNFRSF6B	rs3208008	A	C	0.787356	0.0446397	0.7449552
chr20	62326110	RTEL1	rs3208008	A	C	0.787356	0.0446397	0.7449552
chr19	9006749	MUC16	rs75444444	C	T	0.189655	0.0447431	0.7449552
chr11	5510540	OR52D1	rs190320444	G	GGGCT	0.172414	0.044892	0.7449552
chr11	5510540	OR52D1	rs146317894	G	GGGCT	0.172414	0.044892	0.7449552
chr6	28963248	ZNF311	rs6456880	T	G	0.425287	0.044948	0.7449552
chr22	30975861	PES1	rs34123894	C	T	0.0632184	0.0449584	0.7449552
chr20	17950545	C20orf72	rs11551768	A	T	0.109195	0.0450794	0.7449552

chr1	11579504	PTCHD2	rs2072993	G	C	0.0977011	0.0451678	0.7449552
chr16	28508892	APOBR	rs34395441	G	A	0.0574713	0.0452497	0.7449552
chr10	29581493	LYZL1	rs3818551	A	C	0.212644	0.0452758	0.7449552
chr10	85991784	LRIT1	rs3814211	T	C	0.0804598	0.0452896	0.7449552
chr1	152975941	SPRR3	rs1055935	C	G	0.517241	0.0453244	0.7449552
chrX	6975782	HDHD1	rs868756	C	G	0.494253	0.0454416	0.7449552
chr7	2645552	IQCE	rs2293407	A	G	0.333333	0.0454703	0.7449552
chr6	29141632	OR2J2	rs3116855	T	C	0.517241	0.0455555	0.7449552
chr2	228493211	C2orf83	rs28739019	G	A	0.258621	0.0455634	0.7449552
chr11	55339829	OR4C16	rs557590	A	G	0.91954	0.0455634	0.7449552
chr1	180905448	KIAA1614	rs3795504	G	T	0.436782	0.0455864	0.7449552
chr5	80168937	MSH3	rs26279	G	A	0.729885	0.0456176	0.7449552
chr3	142078759	XRN1	rs73238159	C	T	0.155172	0.0456751	0.7449552
chr19	4217576	ANKRD24	rs150106684	G	C	0.0804598	0.0456904	0.7449552
chr3	158520011	MFSD1	rs28364680	C	T	0.0517241	0.0457106	0.7449552
chr19	43773532	PSG9	rs8101191	G	C	0.0574713	0.0458151	0.7449552
chr7	2644519	IQCE	rs2293404	C	T	0.327586	0.0458207	0.7449552
chr16	3367229	ZNF75A	rs17611866	T	C	0.229885	0.0458469	0.7449552
chr18	30804758	C18orf34	rs457896	A	C	0.12069	0.0458525	0.7449552
chr5	102894673	NUDT12	rs34468716	T	C	0.0862069	0.0459906	0.7449552
chr2	47251469	TTC7A	rs2304290	G	C	0.114943	0.0461277	0.7449552
chr1	17312743	ATP13A2	rs3170740	C	T	0.448276	0.0461998	0.7449552
chr10	115439640	CASP7	rs10553596	CTT	C	0.235632	0.0462589	0.7449552
chr22	31491332	SMTN	rs5997872	C	T	0.126437	0.0463015	0.7449552
chr5	113698631	KCNN2	rs151038013	T	TGCC	0.396552	0.0463473	0.7449552
chr14	60585131	C14orf135	rs308998	A	G	0.103448	0.046426	0.7449552
chr14	60591887	C14orf135	rs167437	G	A	0.103448	0.046426	0.7449552
chr14	60582053	C14orf135	rs150688	G	A	0.896552	0.0464423	0.7449552
chr14	24883887	NYNRIN	rs8017377	G	A	0.494253	0.0464517	0.7449552
chr19	33878837	PEPD	rs17570	G	A	0.183908	0.0465259	0.7449552
chr4	185655192	MLF1IP	rs876839	G	C	0.0632184	0.0465376	0.7449552

chr19	57723007	ZNF264	rs2074858	G	C	0.58046	0.0465882	0.7449552
chr19	57723013	ZNF264	rs917340	G	A	0.58046	0.0465882	0.7449552
chr12	32134815	C12orf35	rs16919122	G	A	0.126437	0.0465916	0.7449552
chr10	96954298	C10orf129	rs591157	A	G	0.557471	0.0466223	0.7449552
chr6	33141280	COL11A2	rs2855430	G	A	0.0977011	0.0466465	0.7449552
chr2	73492614	FBXO41	rs526106	A	T	0.252874	0.0466896	0.7449552
chr2	29240783	FAM179A	rs1109758	A	G	0.281609	0.0467111	0.7449552
chr19	55823344	BRSK1	rs12978445	G	A	0.149425	0.0467163	0.7449552
chr19	53994802	ZNF813	rs10422163	A	T	0.126437	0.0467244	0.7449552
chr5	137500665	BRD8	rs11750814	G	A	0.189655	0.0467619	0.7449552
chr17	7592168	WRAP53	rs2287499	C	G	0.091954	0.0467816	0.7449552
chr21	30925928	GRIK1	rs363504	A	G	0.0689655	0.0468055	0.7449552
chr16	67876823	THAP11	rs377516180	ACAG	A	0.137931	0.046896	0.7449552
chr2	219544388	STK36	rs1863703	A	G	0.0574713	0.0469176	0.7449552
chr2	219553468	STK36	rs16859180	C	T	0.0574713	0.0469176	0.7449552
chr2	219563602	STK36	rs12993599	G	A	0.0574713	0.0469176	0.7449552
chr19	9968434	OLFM2	rs2303100	C	T	0.58046	0.0469208	0.7449552
chr1	3669205	CCDC27	rs10910021	C	G	0.367816	0.0469407	0.7449552
chr19	35434448	ZNF30	rs62122088	A	G	0.132184	0.0471085	0.7449552
chr1	204966428	NFASC	rs2802808	C	G	0.252874	0.0471192	0.7449552
chr11	129722553	TMEM45B	rs558813	C	T	0.183908	0.0471798	0.7449552
chr4	151773593	LRBA	rs1782360	G	C	0.0517241	0.0472316	0.7449552
chr12	10251445	CLEC1A	rs2306894	C	G	0.844828	0.0473331	0.7449552
chr1	247769752	OR2G3	rs61730407	A	G	0.402299	0.0473454	0.7449552
chr12	53044267	KRT2	rs638043	C	T	0.149425	0.0473704	0.7449552
chr20	57768607	ZNF831	rs181984	G	C	0.0517241	0.0474287	0.7449552
chr4	38138856	TBC1D1	rs13110318	G	A	0.103448	0.0474932	0.7449552
chr16	5139186	FAM86A	rs12928528	G	C	0.58046	0.047615	0.7449552
chr16	624114	PIGQ	rs2071979	A	G	0.367816	0.0476641	0.7449552
chr16	633125	PIGQ	rs1045277	T	C	0.367816	0.0476641	0.7449552
chr11	56185224	OR5R1	rs12785840	A	G	0.109195	0.0476757	0.7449552

chr10	96602623	CYP2C19	rs3758581	G	A	0.0574713	0.0478003	0.7449552
chr20	33874720	FAM83C	rs2425049	C	T	0.149425	0.0478125	0.7449552
chr3	66287056	SLC25A26	rs146159281	G	A	0.350575	0.0478352	0.7449552
chr19	44652954	ZNF234	rs2293587	G	A	0.344828	0.0478359	0.7449552
chr13	47243196	LRCH1	rs41284185	C	G	0.0862069	0.0478513	0.7449552
chr16	76532583	CNTNAP4	rs12933808	A	G	0.885057	0.0478671	0.7449552
chr20	42355169	GTSF1L	rs17826038	G	C	0.0517241	0.0478742	0.7449552
chr2	98736225	VWA3B	rs2305355	C	T	0.0517241	0.0479301	0.7449552
chr2	95947085	PROM2	rs12992066	A	G	0.724138	0.0479438	0.7449552
chr4	144621779	FREM3	rs55935372	A	G	0.350575	0.0479581	0.7449552
chr19	51728641	CD33	rs2455069	A	G	0.431034	0.0479844	0.7449552
chr8	143922620	GML	rs3764795	C	T	0.0632184	0.0481269	0.7449552
chr11	308314	IFITM2	rs14408	T	C	0.431034	0.0481349	0.7449552
chr21	43808627	TMPRSS3	rs35227181	C	T	0.132184	0.0481741	0.7449552
chr4	187630590	FAT1	rs3733415	G	A	0.172414	0.048257	0.7449552
chr19	9968139	OLFM2	rs11556087	G	A	0.241379	0.0482818	0.7449552
chr22	39134207	SUN2	rs2072797	C	T	0.103448	0.0483067	0.7449552
chr10	115377290	NRAP	rs77678145	T	C	0.0977011	0.0483463	0.7449552
chr8	17396415	SLC7A2	rs13259978	G	C	0.201149	0.0483945	0.7449552
chr19	51323473	KLK1	rs5516	C	G	0.632184	0.0485341	0.7449552
chr14	105406372	AHNAK2	rs61421370	C	T	0.45977	0.0485484	0.7449552
chr14	105409907	AHNAK2	rs10141053	T	C	0.45977	0.0485484	0.7449552
chr12	122186317	TMEM120B	rs28655666	G	A	0.574713	0.048601	0.7449552
chr9	112219474	PTPN3	rs3793524	C	G	0.344828	0.0486978	0.7449552
chr19	56703248	ZSCAN5B	rs527025	G	A	0.356322	0.048755	0.7449552
chr9	96021312	WNK2	rs10761203	G	A	0.505747	0.0487586	0.7449552
chr1	79093818	IFI44L	rs273259	A	G	0.281609	0.0488657	0.7449552
chr17	46847364	TTLL6	rs2032844	C	A	0.270115	0.0488664	0.7449552
chr11	5602929	OR52B6	rs74053516	C	A	0.0804598	0.048917	0.7449552
chr3	14745857	C3orf20	rs17040196	G	A	0.482759	0.0489597	0.7449552
chr11	116691675	APOA4	rs675	T	A	0.201149	0.0489758	0.7449552

chr1	119469188	TBX15	rs10494217	G	T	0.206897	0.0490163	0.7449552
chr19	38202515	ZNF607	rs12461753	G	A	0.16092	0.04902	0.7449552
chr5	140801104	PCDHGA11	rs11167744	T	C	0.103448	0.0490641	0.7449552
chr5	140803241	PCDHGA11	rs57195665	C	A	0.103448	0.0490641	0.7449552
chr5	140772898	PCDHGA8	rs3214276	GC	G	0.103448	0.0490641	0.7449552
chr19	43087484	CEACAM8	rs1126458	A	C	0.126437	0.0491087	0.7449552
chr19	37310344	ZNF790	rs3745775	T	C	0.293103	0.0491277	0.7449552
chr5	140595343	PCDHB13	rs56309578	G	C	0.091954	0.0491448	0.7449552
chr7	140158851	MKRN1	rs2272095	C	G	0.241379	0.0492204	0.7449552
chr7	107720162	LAMB4	rs9690688	C	A	0.0632184	0.0492789	0.7449552
chr6	38750888	DNAH8	rs3823430	A	G	0.12069	0.0492867	0.7449552
chr5	148206473	ADRB2	rs1042714	G	C	0.603448	0.0493142	0.7449552
chr17	78337058	RNF213	rs35332090	G	C	0.0747126	0.0493574	0.7449552
chr16	3640274	SLX4	rs714181	G	A	0.0632184	0.0493574	0.7449552
chr3	185990096	DGKG	rs2193587	C	T	0.775862	0.0494689	0.7449552
chr14	75248652	YLPM1	rs45617140	C	G	0.109195	0.049531	0.7449552
chr6	29080450	OR2J3	rs3130765	G	A	0.821839	0.0495359	0.7449552
chr4	87770252	SLC10A6	rs17694522	G	A	0.0517241	0.0496464	0.7449552
chr19	43234049	PSG3	rs28698193	A	T	0.114943	0.0497647	0.7449552
chr19	43268235	PSG8	rs75257969	A	C	0.114943	0.0497647	0.7449552
chr1	9009406	CA6	rs2274327	C	T	0.5	0.049778	0.7449552
chr2	219895548	CCDC108	rs56411706	C	A	0.0804598	0.0497915	0.7449552
chr2	219900068	CCDC108	rs17852959	C	T	0.0804598	0.0497915	0.7449552
chr3	46620614	TDGF1	rs11130097	T	C	0.505747	0.0499125	0.7449552
chr12	70091452	BEST3	rs1025016	A	G	0.0747126	0.0499257	0.7449552
chr5	68695940	RAD17	rs1045051	T	G	0.310345	0.0499499	0.7449552
