# Supplementary material for: Tensin 1 (TNS1) is a modifier gene for low body mass index (BMI) in homozygous [F508del]CFTR patients
Source: Physiol Rep. 2021 Jun 4;9(11):e14886. doi: 10.14814/phy2.14886 (PMC8176904; doi:10.14814/phy2.14886)
Supplement: Supplementary file 6 — Table S4 [file PHY2-9-e14886-s004.pdf]

Table SI4: Phenotypic comparisons of CF cohort and their TNS1 genotype
BMI Comparison of TNS1 Genotypes

CF Cohort n=87						
	TNS1 (rs918949)	n1	BMI	Reference TNS1 (rs918949)	n2	P
	C/C	11	23.6	T/T	38	0.16
	C/T	38	22.7	C/C	11	0.61
	T/T	38	22.0	C/T	38	0.3
Homozygous [*F508del*] n=45						
	TNS1 (rs918949)	n1	BMI	Reference TNS1 (rs918949)	n2	P
	C/C	2	25.4	T/T	29	NA
	C/T	14	23.0	C/C	2	NA
	T/T	29	21.6	C/T	14	0.023
Compound Heterozygous [*F508del*] n=42						
	TNS1 (rs918949)	n1	BMI	Reference TNS1 (rs918949)	n2	P
	C/C	9	23.2	T/T	9	0.63
	C/T	24	22.6	C/C	9	0.12
	T/T	9	23.1	C/T	24	0.12
CF Cohort n=87						
	TNS1 (rs2571445)	n1	BMI	Reference TNS1 (rs2571445)	n2	P
	A/A	11	23.6	G/G	37	0.16
	A/G	39	22.7	A/A	11	0.63
	G/G	37	21.9	A/G	39	0.32
Homozygous [*F508del*] n=45						
	TNS1 (rs2571445)	n1	BMI	Reference TNS1 (rs2571445)	n2	P
	A/A	2	25.4	G/G	28	NA
	A/G	15	23.0	A/A	2	NA
	G/G	28	21.6	A/G	15	0.02
Compound Heterozygous [*F508del*] n=42						
	TNS1 (rs2571445)	n1	BMI	Reference TNS1 (rs2571445)	n2	P
	A/A	9	23.2	G/G	9	0.63
	A/G	24	22.6	A/A	9	0.12
	G/G	9	23.1	A/G	24	0.12

ppFEV1 Comparison of TNS1 Genotypes

CF Cohort n=87

TNS1 (rs918949)	n1	ppFEV1	Reference TNS1 (rs918949)	n2	P
C/C	11	65.8	T/T	38	0.11
C/T	38	61.7	C/C	11	0.43
T/T	38	56	C/T	38	0.27

Homozygous [*F508del*] n=45

TNS1 (rs918949)	n1	ppFEV1	Reference TNS1 (rs918949)	n2	P
C/C	2	75.2	T/T	29	NA
C/T	14	60.4	C/C	2	NA
T/T	29	57.1	C/T	14	0.73

Compound Heterozygous [*F508del*] n=42

TNS1 (rs918949)	n1	ppFEV1	Reference TNS1 (rs918949)	n2	P
C/C	9	63.7	T/T	9	0.24
C/T	24	62.5	C/C	9	0.31
T/T	9	52.6	C/T	24	0.54

CF Cohort n=87

TNS1 (rs2571445)	n1	ppFEV1	Reference TNS1 (rs2571445)	n2	P
A/A	11	65.8	G/G	37	0.11
A/G	39	62.4	A/A	11	0.48
G/G	37	55.2	A/G	39	0.2

Homozygous [*F508del*] n=45

TNS1 (rs2571445)	n1	ppFEV1	Reference TNS1 (rs2571445)	n2	P
A/A	2	75.2	G/G	28	NA
A/G	15	62.2	A/A	2	NA
G/G	28	56	A/G	15	0.73

Compound Heterozygous [*F508del*] n=42

TNS1 (rs2571445)	n1	ppFEV1	Reference TNS1 (rs2571445)	n2	P
A/A	9	63.7	G/G	9	0.24
A/G	24	62.5	A/A	9	0.54
G/G	9	52.6	A/G	24	0.54

ppFVC Comparison of TNS1 Genotypes

CF Cohort n=87

TNS1 (rs918949)	n1	ppFVC	Reference TNS1 (rs918949)	n2	P
C/C	11	86.2	T/T	38	0.07
C/T	38	78.5	C/C	11	0.13
T/T	38	74.5	C/T	38	0.59

Homozygous [*F508del*] n=45

TNS1 (rs918949)	n1	ppFVC	Reference TNS1 (rs918949)	n2	P
C/C	2	100.2	T/T	29	NA
C/T	14	78.9	C/C	2	NA
T/T	29	76.3	C/T	14	0.85

Compound Heterozygous [*F508del*] n=42

TNS1 (rs918949)	n1	ppFVC	Reference TNS1 (rs918949)	n2	
C/C	9	83.1	T/T	9	0.15
C/T	24	78.3	C/C	9	0.74
T/T	9	68.6	C/T	24	0.74

CF Cohort n=87

TNS1 (rs2571445)	n1	ppFVC	Reference TNS1 (rs2571445)	n2	P
A/A	11	86.2	G/G	37	0.07
A/G	39	79.2	A/A	11	0.17
G/G	37	73.6	A/G	39	0.43

Homozygous [*F508del*] n=45

TNS1 (rs2571445)	n1	ppFVC	Reference TNS1 (rs2571445)	n2	P
A/A	2	100.2	G/G	28	NA
A/G	15	80.6	A/A	2	NA
G/G	28	75.2	A/G	15	0.77

Compound Heterozygous [*F508del*] n=42

TNS1 (rs2571445)	n1	ppFVC	Reference TNS1 (rs2571445)	n2	P
A/A	9	83.1	G/G	9	0.15
A/G	24	78.3	A/A	9	0.74
G/G	9	68.6	A/G	24	0.74

Sex Comparison of TNS1 Genotypes

CF Cohort n=87

TNS1 (rs918949)	n1	Sex (M/F)	Reference TNS1 (rs918949)	n2	P
C/C	11	7/4	T/T	38	0.34
C/T	38	18/20	C/C	11	0.31
T/T	38	19/19	C/T	38	0.74

Homozygous [*F508del*] n=45

TNS1 (rs918949)	n1	Sex (M/F)	Reference TNS1 (rs918949)	n2	P
C/C	2	2/0	T/T	29	NA
C/T	14	6/8	C/C	2	NA
T/T	29	13/16	C/T	14	0.83

Compound Heterozygous [*F508del*] n=42

TNS1 (rs918949)	n1	Sex (M/F)	Reference TNS1 (rs918949)	n2	P
C/C	9	5/4	T/T	9	0.98
C/T	24	12/12	C/C	9	0.73
T/T	9	6/3	C/T	24	0.73

CF Cohort n=87

TNS1 (rs2571445)	n1	Sex (M/F)	Reference TNS1 (rs2571445)	n2	P
A/A	11	7/4	G/G	37	0.34
A/G	39	19/20	A/A	11	0.37
G/G	37	19/19	A/G	39	0.99

Homozygous [*F508del*] n=45

TNS1 (rs2571445)	n1	Sex (M/F)	Reference TNS1 (rs2571445)	n2	P
A/A	2	2/0	G/G	28	NA
A/G	15	7/8	A/A	2	NA
G/G	28	12/16	A/G	15	0.78

Compound Heterozygous [*F508del*] n=42

TNS1 (rs2571445)	n1	Sex (M/F)	Reference TNS1 (rs2571445)	n2	P
A/A	9	5/4	G/G	9	0.98
A/G	24	12/12	A/A	9	0.73
G/G	9	6/3	A/G	24	0.73

CF Related Diabetes Comparison of TNS1 Genotypes

	TNS1 (rs918949)	n1	Diabetes (Y/N)	Reference TNS1 (rs918949)	n2	P
CF Cohort n=87						
	C/C	11	2/9	T/T	38	0.12
	C/T	38	9/29	C/C	11	0.83
	T/T	38	16/22	C/T	38	0.11
Homozygous [*F508del*] n=45						
	TNS1 (rs918949)	n1	Diabetes (Y/N)	Reference TNS1 (rs918949)	n2	P
	C/C	2	1/1	T/T	29	NA
	C/T	14	6/8	C/C	2	NA
	T/T	29	13/16	C/T	14	0.87
Compound Heterozygous [*F508del*] n=42						
	TNS1 (rs918949)	n1	Diabetes (Y/N)	Reference TNS1 (rs918949)	n2	P
	C/C	9	1/8	T/T	9	0.53
	C/T	24	3/21	C/C	9	0.37
	T/T	9	3/6	C/T	24	0.37
CF Cohort n=87						
	TNS1 (rs2571445)	n1	Diabetes (Y/N)	Reference TNS1 (rs2571445)	n2	P
	A/A	11	2/9	G/G	37	0.12
	A/G	39	9/30	A/A	11	0.87
	G/G	37	16/21	A/G	39	0.08
Homozygous [*F508del*] n=45						
	TNS1 (rs2571445)	n1	Diabetes (Y/N)	Reference TNS1 (rs2571445)	n2	P
	A/A	2	1/1	G/G	28	NA
	A/G	15	6/9	A/A	2	NA
	G/G	28	13/15	A/G	15	0.72
Compound Heterozygous [*F508del*] n=42						
	TNS1 (rs2571445)	n1	Diabetes (Y/N)	Reference TNS1 (rs2571445)	n2	P
	A/A	9	1/8	G/G	9	0.53
	A/G	24	3/21	A/A	9	0.37
	G/G	9	3/6	A/G	24	0.37

Pancreatic Sufficiency Comparison of TNS1 Genotypes

CF Cohort n=87

TNS1 (rs918949)	n1	Pancreatic Sufficiency (PI/PS)	Reference TNS1 (rs918949)	n2	P
C/C	11	8/3	T/T	38	0.21
C/T	38	30/8	C/C	11	0.33
T/T	38	33/5	C/T	38	0.54

Homozygous [*F508del*] n=45

TNS1 (rs918949)	n1	Pancreatic Sufficiency (PI/PS)	Reference TNS1 (rs918949)	n2	P
C/C	2	1/1	T/T	29	NA
C/T	14	13/1	C/C	2	NA
T/T	29	27/2	C/T	14	0.9

Compound Heterozygous [*F508del*] n=42

TNS1 (rs918949)	n1	Pancreatic Sufficiency (PI/PS)	Reference TNS1 (rs918949)	n2	P
C/C	9	7/2	T/T	9	0.31
C/T	24	17/7	C/C	9	0.49
T/T	9	6/3	C/T	24	0.49

CF Cohort n=87

TNS1 (rs2571445)	n1	Pancreatic Sufficiency (PI/PS)	Reference TNS1 (rs2571445)	n2	P
A/A	11	8/3	G/G	37	0.21
A/G	39	31/8	A/A	11	0.32
G/G	37	32/5	A/G	39	0.55

Homozygous [*F508del*] n=45

TNS1 (rs2571445)	n1	Pancreatic Sufficiency (PI/PS)	Reference TNS1 (rs2571445)	n2	P
A/A	2	1/1	G/G	28	NA
A/G	15	14/1	A/A	2	NA
G/G	28	26/2	A/G	15	0.89

Compound Heterozygous [*F508del*] n=42

TNS1 (rs2571445)	n1	Pancreatic Sufficiency (PI/PS)	Reference TNS1 (rs2571445)	n2	P
A/A	9	7/2	G/G	9	0.31
A/G	24	17/7	A/A	9	0.49
G/G	9	6/3	A/G	24	0.49
